# Multibubble Sonoluminescence from a Theoretical Perspective

**DOI:** 10.3390/molecules26154624

**Published:** 2021-07-30

**Authors:** Kyuichi Yasui

**Affiliations:** National Institute of Advanced Industrial Science and Technology (AIST), Nagoya 463-8560, Japan; k.yasui@aist.go.jp

**Keywords:** vaporous bubble, gaseous bubble, OH chemiluminescence, plasma, ionization potential lowering, Na-line emission, bubble–bubble interaction, acoustic field, sulfuric acid, applications

## Abstract

In the present review, complexity in multibubble sonoluminescence (MBSL) is discussed. At relatively low ultrasonic frequency, a cavitation bubble is filled mostly with water vapor at relatively high acoustic amplitude which results in OH-line emission by chemiluminescence as well as emissions from weakly ionized plasma formed inside a bubble at the end of the violent bubble collapse. At relatively high ultrasonic frequency or at relatively low acoustic amplitude at relatively low ultrasonic frequency, a cavitation bubble is mostly filled with noncondensable gases such as air or argon at the end of the bubble collapse, which results in relatively high bubble temperature and light emissions from plasma formed inside a bubble. Ionization potential lowering for atoms and molecules occurs due to the extremely high density inside a bubble at the end of the violent bubble collapse, which is one of the main reasons for the plasma formation inside a bubble in addition to the high bubble temperature due to quasi-adiabatic compression of a bubble, where “quasi” means that appreciable thermal conduction takes place between the heated interior of a bubble and the surrounding liquid. Due to bubble–bubble interaction, liquid droplets enter bubbles at the bubble collapse, which results in sodium-line emission.

## 1. Introduction

**Figure 1:**
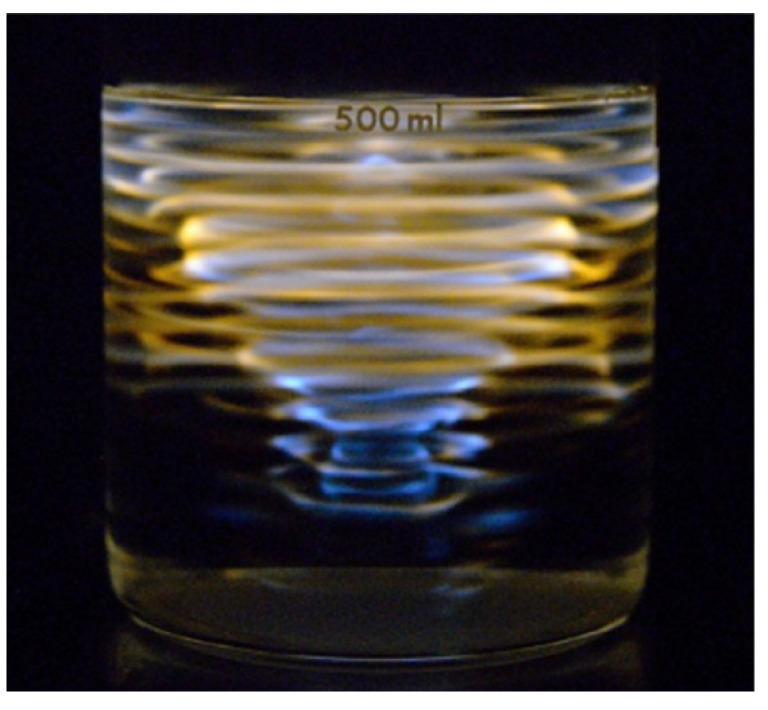
Photographic image of MBSL from Ar-saturated SDS solution at an acoustic power of 4 W [3]. Ultrasound at a frequency of 148 kHz was irradiated from the bottom of the beaker. Copyright 2015, with the permission from Elsevier.

Multibubble sonoluminescence (MBSL) is the light emission phenomenon from cavitation bubbles in liquid irradiated by strong ultrasound (Figure 1) [1,2,3]. Cavitation is the appearance of bubbles in liquid by strong decrease in local instantaneous pressure by strong ultrasound or by some hydrodynamic motion such as that around a ship propeller [4]. In cavitation, a bubble collapses very violently after bubble expansion under some conditions. There are two reasons for the violent bubble collapse [5]. One is the spherical geometry of a bubble collapse. According to the continuity of the liquid, the speed of the bubble collapse increases as the bubble radius decreases because the surface area of a bubble decreases. The other is the inertia of the surrounding liquid. Due to the inertia of the ingoing liquid, the bubble collapse becomes very violent. The violent bubble collapse is called Rayleigh collapse [5,6].

At the end of the violent bubble collapse, temperature and pressure inside a bubble significantly increase to more than 4000 K and 300 bar (1 bar = 10^5^ Pa = 0.987 atm), respectively [7,8], due to quasi-adiabatic compression of a bubble, where “quasi-“ means the appreciable amount of thermal conduction takes place between the heated interior of a bubble and the surrounding liquid. As a result, water vapor inside a bubble is thermally dissociated and OH radicals are formed, which is called sonochemical reactions [5,9]. Furthermore, faint light of sonoluminescence (SL) is emitted from a bubble. Duration of high temperature and pressure inside a bubble is only less than 1–10 ns. Thus, the SL pulse width from a bubble is less than 1 ns [1,6,10].

MBSL was discovered in 1933 by Marinesco and Trillat [11] who observed the fogging of the photographic plate immersed in the liquid irradiated by ultrasound. MBSL was recognized as the light emission from the cavitating liquid in 1934 by Frenzel and Schultes [12]. Significant development in SL research has been brought about after the discovery of single-bubble sonoluminescence (SBSL) in 1990 by Gaitan and Crum [13] (The first report on SBSL was in 1962 by Yosioka and Omura [14]. However, the work was not confirmed.) SBSL is SL from a stably pulsating bubble trapped at pressure antinode of a standing ultrasonic wave [1,2,6]. In 1991, Barber and Putterman [15] reported in Nature that SBSL pulse width is less than 100 ps, which attracted many researchers’ attention. After that, there has been significant development in SL research. Before the discovery of SBSL in 1990, MBSL, which was simply called SL, had been considered to originate mainly in the recombination of free radicals inside a bubble [16]. The development of the SBSL research has been reviewed by Brenner, Hilgenfeldt, and Lohse [6].

Another significant finding in SL research is the evidence of (thermal) plasma formation inside a bubble in sulfuric acid in optical spectra of SBSL by Flannigan and Suslick in 2005 [17].

In the present review, complexity in MBSL is discussed based on the understanding developed after the discovery of SBSL. Furthermore, unsolved problems in MBSL are also discussed.

## 2. Theoretical Model

Firstly, model of bubble dynamics developed in studies of SBSL is reviewed. Some bubbles in MBSL are nearly isolated and spherical, which are similar to SBSL bubbles [18]. Furthermore, bubble dynamics model developed for SBSL can be modified for MBSL bubbles taking into account the effect of bubble–bubble interaction [19].

There are two types in models of bubble dynamics for SBSL [2]. One is the shock wave model that a spherical shock wave converges at the bubble center where extremely high temperature plasma is formed. The other is the hot-spot model that nearly the whole bubble is heated by quasi-adiabatic compression, where ‘quasi-‘ means appreciable thermal conduction takes place between the heated bubble interior and the surrounding liquid.

Numerical simulations of bubble collapse by fundamental equations of fluid dynamics have revealed that temperature and pressure are nearly spatially uniform inside a bubble and that there is no shock wave formation inside a bubble under many conditions [20]. The reason for no shock formation is as follows [21]. As the temperature near the bubble wall is lower than that near the bubble center due to thermal conduction between the heated interior of a bubble and the surrounding liquid, sound speed is lower near the bubble wall. For the shock wave formation, a pressure wave radiated inwardly from the bubble wall should overtake previously radiated pressure waves which move with the sound speed plus radial fluid (gas) velocity. Typical bubble collapse is not so violent to overcome the adverse gradient of sound speed for the shock formation. Thus, no shock wave is formed inside a collapsing bubble under many conditions.

**Figure 2:**
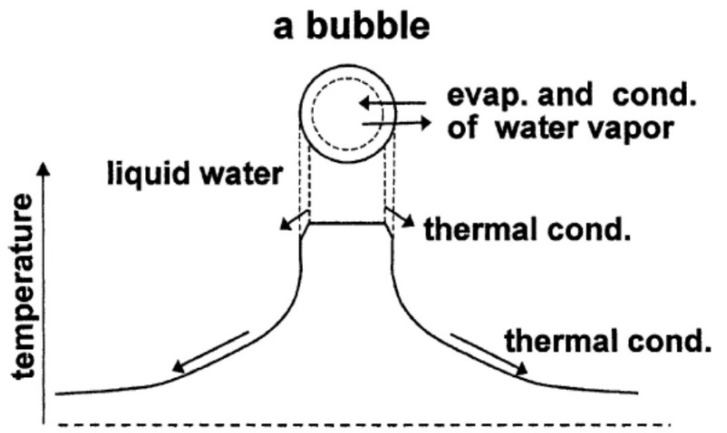
The model of bubble dynamics [22]. Copyright 2004, with the permission from Elsevier.

Thus, in the present review, the hot-spot model is discussed (Figure 2) [5,22,23,24,25,26]. Temperature is assumed to be spatially uniform inside a bubble except at the thermal boundary layer near the bubble wall. Thermal conduction takes place both inside and outside a bubble. Non-equilibrium evaporation and condensation of water vapor takes place at the bubble wall.

The radius-time curve of a bubble in liquid irradiated with ultrasound is numerically calculated by the Keller equation [5].

(1) $$\left(1-\frac{\dot{R}}{c_{\infty }}\right)R\ddot{R}+\frac{3}{2}{\dot{R}}^{2}\left(1-\frac{\dot{R}}{3c_{\infty }}\right)=\frac{1}{\rho_{L,\infty }}\left(1+\frac{\dot{R}}{c_{\infty }}\right)\left[p_{B}-p_{s}\left(t\right)-p_{\infty }\right]+\frac{R}{c_{\infty }\rho_{L,\infty }}\frac{dp_{B}}{dt}$$

where *R* is bubble radius, dot denotes the time derivative (d/dt), $c_{\infty }$ is the sound speed in the liquid far from a bubble, $\rho_{L,\infty }$ is the liquid density far from a bubble, $p_{B}$ is the liquid pressure on the external side of the bubble wall, $p_{s}\left(t\right)$ is a nonconstant ambient pressure component such as a sound field ($p_{s}\left(t\right)=-A\sin\omega t$, where A is the acoustic amplitude and ω is angular frequency of ultrasound), and $p_{\infty }$ is the undisturbed pressure. The method of numerical simulations is simply as follows [5].

(2) $$R\left(t+\Delta t\right)=R\left(t\right)+\dot{R}\left(t\right)\Delta t$$

(3) $$\dot{R}\left(t+\Delta t\right)=\dot{R}\left(t\right)+\ddot{R}\left(t\right)\Delta t$$

where $\ddot{R}\left(t\right)$ in Equation (3) is calculated by Equation (1).

In the hot-spot model [5,23,24,25,26], the following effects are taken into account; non-equilibrium chemical reactions inside a bubble, variation of liquid temperature at the bubble wall, dependence of physical quantities such as saturated vapor pressure, surface tension, etc. on liquid temperature at the bubble wall, thermal ionization of molecules and atoms inside a bubble including the effect of ionization potential lowering due to high density, and light emissions inside a bubble including chemiluminescence of OH, electron-atom and electron-ion bremsstrahlung, radiative recombination of electrons and ions, radiative attachment of electrons to neutral atoms, etc.

**Figure 3:**
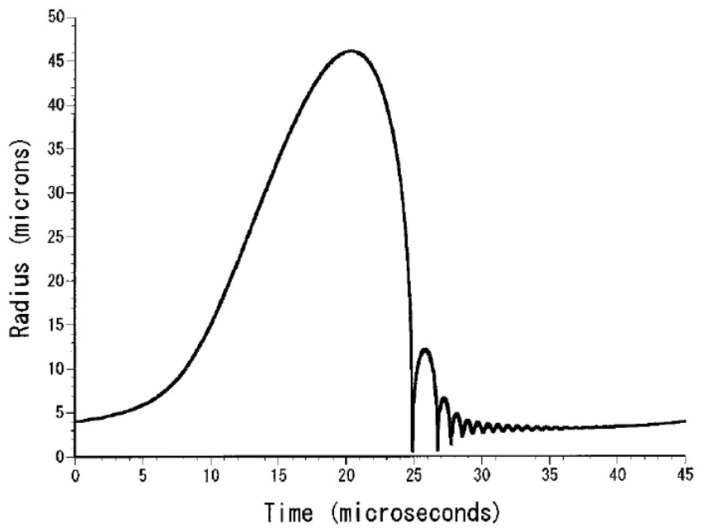
The calculated radius-time curve of an argon bubble in water for one acoustic cycle when the frequency and the amplitude of ultrasound are 22 kHz and 1.32 bar, respectively and the ambient bubble radius is 4 μm [27]. Copyright 2002, with the permission of AIP Publishing.

**Figure 4:**
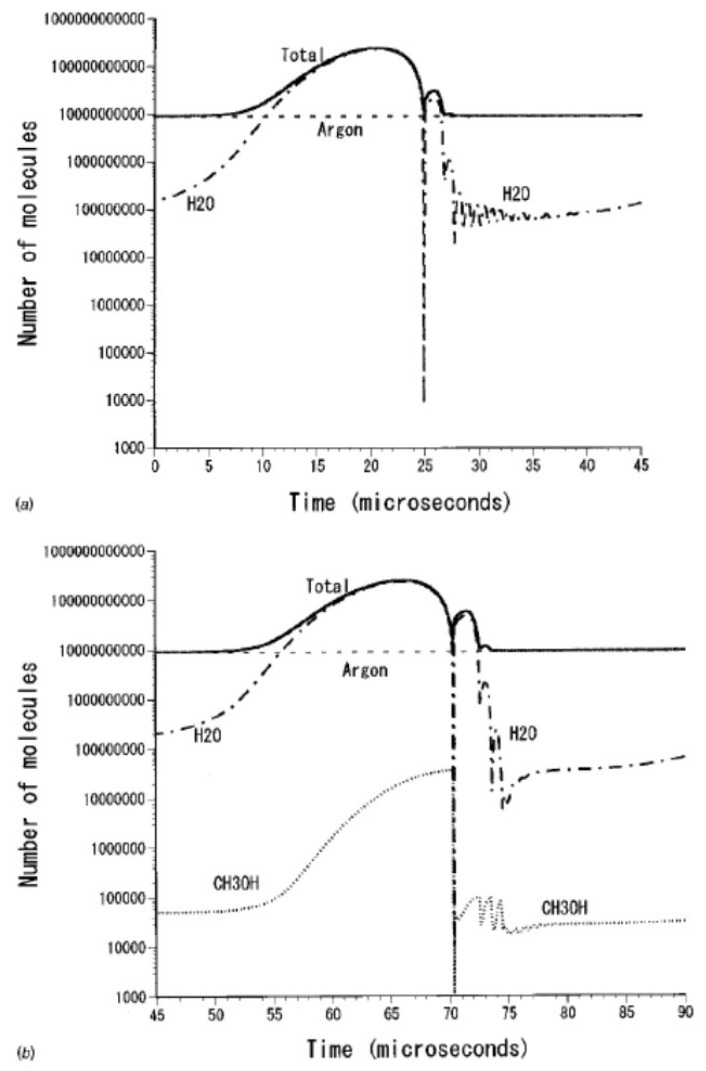
The numbers of molecules inside a bubble as a function of time for one acoustic cycle in water (**a**) and in aqueous methanol solution (**b**) [27]. Copyright 2002, with the permission of AIP Publishing.

**Figure 5:**
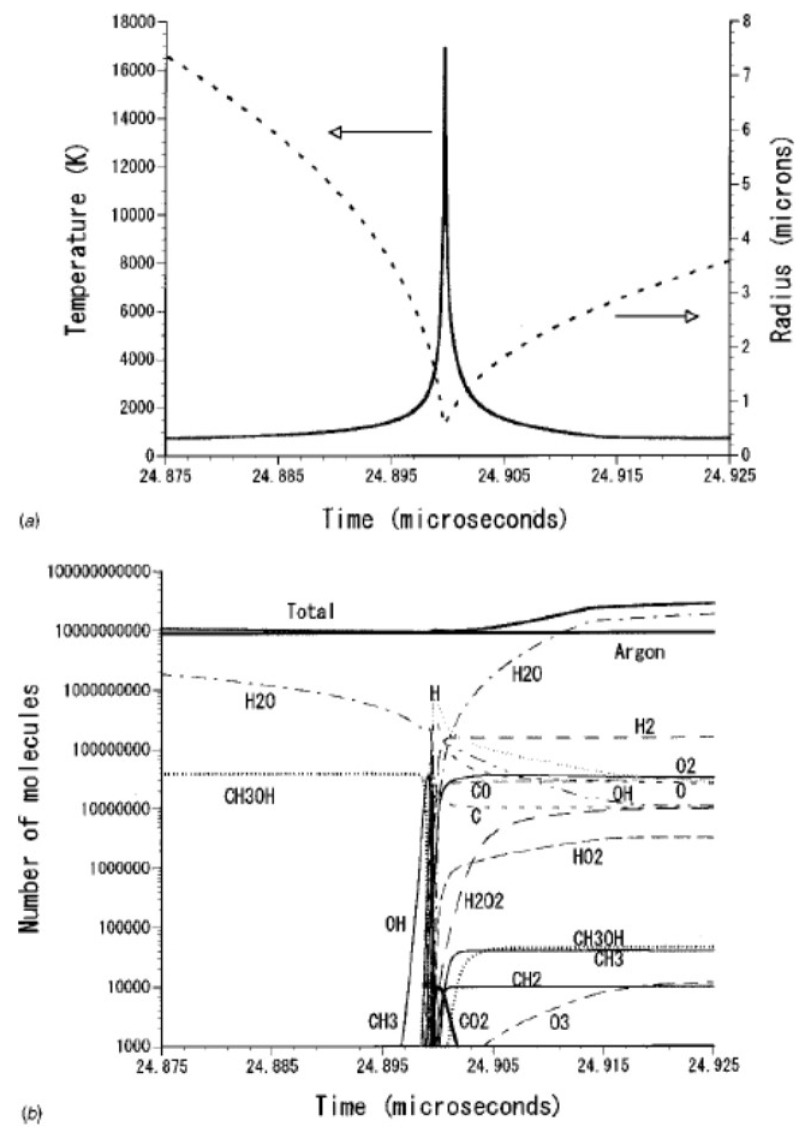
The calculated results at around the minimum bubble radius for aqueous methanol solution [27]. The condition is the same as that of Figure 3. (**a**) The bubble radius and the temperature inside a bubble. (**b**) The numbers of molecules inside a bubble with logarithmic vertical axis. Copyright 2002, with the permission of AIP Publishing.

**Figure 6:**
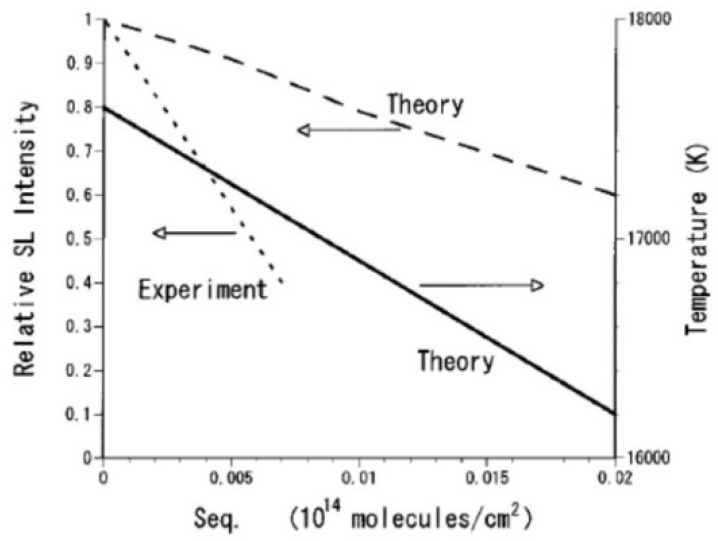
The energy of the emitted light per bubble collapse and the bubble temperature at the collapse as a function of the equilibrium surface concentration of methanol (S_eq_) [27]. The experimental data of the relative sonoluminescence intensity are also shown [30]. Copyright 2002, with the permission of AIP Publishing.

**Figure 7:**
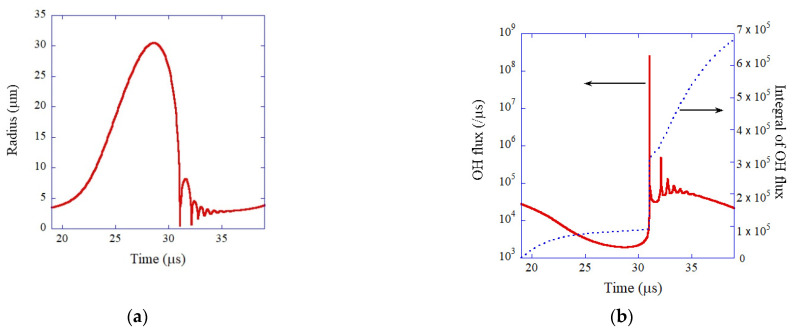
The calculated results under the condition of single bubble sonochemistry [24]. A SBSL bubble with R_0_ = 3.6 μm in a steady state in water at 3 °C is irradiated by an ultrasonic wave of 52 kHz and 1.52 bar. (**a**) The bubble radius (**b**) The dissolution rate of OH radicals into the liquid from the interior of the bubble (solid line) and its time integral (dotted line). Copyright 2005. with the permission of AIP Publishing.

An example of calculated radius-time curve of a SBSL bubble is shown in Figure 3 [27]. A bubble expands during the rarefaction phase of ultrasound. At the compression phase of ultrasound, a bubble collapses violently, which is followed by bouncing motion. The numbers of molecules inside a bubble are shown as a function of time in Figure 4 [27]. In SBSL, nitrogen in an initially air bubble is burned and changes to NO_x_ and HNO_x_ which gradually dissolve into the surrounding liquid water [28,29]. As a result, the bubble content becomes mainly argon (1% of air is argon), which is called argon rectification hypothesis. The argon rectification hypothesis has been validated both experimentally and theoretically [6]. Thus, the calculated results in Figure 3, Figure 4, Figure 5, Figure 6 and Figure 7 for SBSL are for an argon bubble.

As seen in Figure 4, during the bubble expansion, intense evaporation of water vapor into the bubble takes place. As a result, near the maximum bubble radius, the bubble content is mostly water vapor. During the bubble collapse, on the other hand, intense condensation of water vapor occurs at the bubble wall. As a result, the bubble content is mostly argon near the end of the violent bubble collapse. In Figure 4b, the calculated result for aqueous methanol solution is shown. In this case, evaporation of methanol also occurs during the bubble expansion. During the bubble collapse, intense condensation of methanol occurs at the bubble wall as well as that of water vapor [27].

At the end of the violent bubble collapse, temperature inside an argon bubble in aqueous methanol solution under the condition of Figure 3 and Figure 4 increases to 17,000 K as shown in Figure 5a [27]. As a result, water vapor as well as methanol inside a bubble is thermally dissociated as shown in Figure 5b. This kind of reactions are called sonochemical reactions [5].

Due to the endothermic dissociation of methanol inside a bubble, temperature inside a bubble decreases as the methanol concentration increases (Figure 6) [27]. As a result, the intensity of SBSL decreases as the methanol concentration increases, which semi-quantitatively agrees with the experimental data [30]. Theoretically, the SBSL intensity is calculated by the following contributions for light emissions from thermal plasma formed inside a bubble; electron-atom bremsstrahlung, electron-ion bremsstrahlung, radiative recombination of electrons and ions, and radiative attachment of electrons to neutral particles. Electron-atom and electron-ion bremsstrahlung is light emission when electrons are decelerated by collisions with neutral atoms and positive ions, respectively. In general, when a charged particle such as an electron is decelerated, light is emitted, known as bremsstrahlung [31]. Details of the equations for the calculations of the light intensity are described in Ref. [25].

The model of bubble dynamics including non-equilibrium chemical reactions inside a bubble as well as SL light emission has been validated in the study of single-bubble sonochemistry (Figure 7) [24]. In 2002, Didenko and Suslick [32] reported experimentally the production rate of OH radicals from a SBSL bubble as $8.2\times 10^{5}$ molecules per acoustic cycle. They also reported that the number of photons emitted from a SBSL bubble per acoustic cycle is $7.5\times 10^{4}$. OH radicals are produced inside a heated bubble by the dissociation of water vapor (H_2_O). In Figure 7b, the calculated dissolution rate of OH radicals into the liquid from the interior of a SBSL bubble is shown as a function of time for one acoustic cycle as well as its time integral [24]. The simulated total amount of OH radicals dissolved into the liquid from a SBSL bubble is $6.6\times 10^{5}$ molecules per acoustic cycle, which almost agrees with the experimental data [24,32]. Furthermore, the simulated number of photons emitted from a SBSL bubble per pulse is $8.0\times 10^{4}$, which almost agrees with the exprimental data. Thus, the model has been validated.

## 3. Vaporous and Gaseous Bubbles

When the bubble content is mostly water vapor even at the end of the bubble collapse, such bubbles are called vaporous bubbles [33]. On the other hand, when the bubble content at the end of the bubble collapse is noncondensable gas such as air, such bubbles are called gaseous bubbles [33]. In this section, difference in mechanism of MBSL emission between vaporous and gaseous bubbles is discussed [25,34].

**Figure 8:**
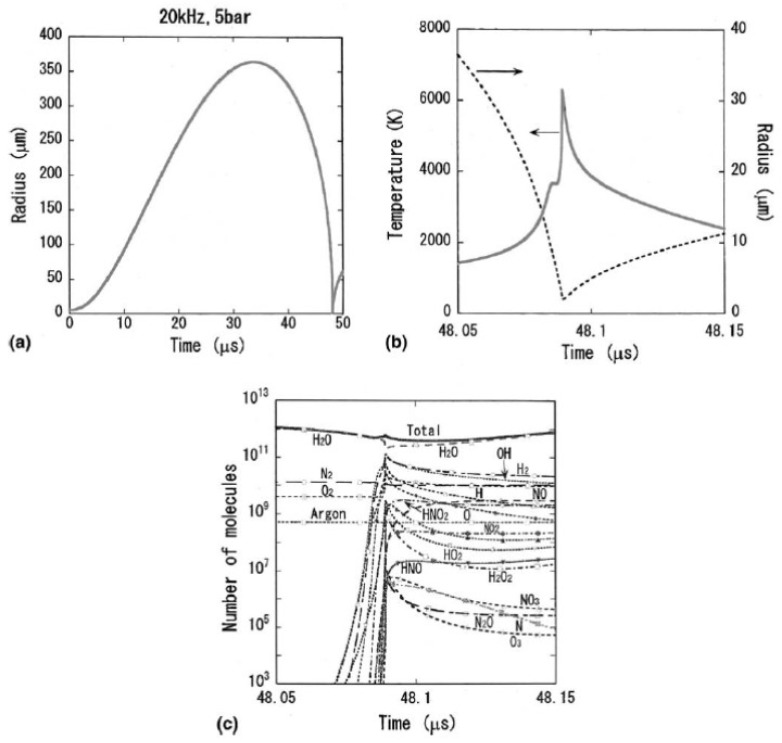
Calculated results for an air bubble in liquid water at 20 °C irradiated by an ultrasonic wave of 20 kHz and 5 bar in frequency and pressure amplitude, respectively [33]. The ambient bubble radius is 5 μm. (**a**) The radius-time curve for one acoustic cycle. A bubble disintegrates into daughter bubbles by shape instability at t = 152.7 μs which is out of this figure. (**b**) The temperature inside a bubble and the bubble radius as a function of time at around the end of the bubble collapse only for 0.1 μs. (**c**) Numbers of molecules of various species inside a bubble as a function of time with the same time axis as that of (**b**). The vertical axis is in logarithmic scale. Copyright 2005, with the permission from Elsevier.

According to numerical simulations, vaporous bubbles appear at relatively high acoustic amplitudes at relatively low ultrasonic frequencies [35]. For example, at 20 kHz and 5 bar in ultrasonic frequency and pressure amplitude, respectively, a bubble expands dramatically during the rarefaction phase of ultrasound (Figure 8a) [33]. As a result, intense evaporation of water takes place during the bubble expansion into a bubble in order to maintain the saturated vapor pressure inside a bubble. Although intense condensation of water vapor takes place at the bubble wall at the bubble collapse, still the main bubble content is water vapor even at the end of the bubble collapse (Figure 8c) [33]. It should be noted that condensation at the bubble collapse is strongly in non-equilibrium, and the partial pressure of water vapor inside a bubble is several orders of magnitude larger than the saturated vapor pressure [36].

The peak bubble temperature in a vaporous air bubble in 20 °C water is always about 6300 K due to the following reason (Figure 8b) [33,34]. As the bubble temperature increases, water vapor is gradually dissociated mainly by H_2_O + M → OH + H + M and H_2_O + H → OH + H_2_, where M is the third body. The endothermic vapor dissociation suppresses the further temperature increase. Thus, the bubble temperature remains nearly constant (~4000 K) by this effect just before the end of the bubble collapse (Figure 8b). As the speed of the bubble collapse further increases, the endothermic heat of vapor dissociation is no longer able to keep the bubble temperature constant. This condition is similar for any case, which results in the nearly constant peak temperature inside a bubble at relatively high acoustic amplitudes [34]. This is discussed again later.

**Figure 9:**
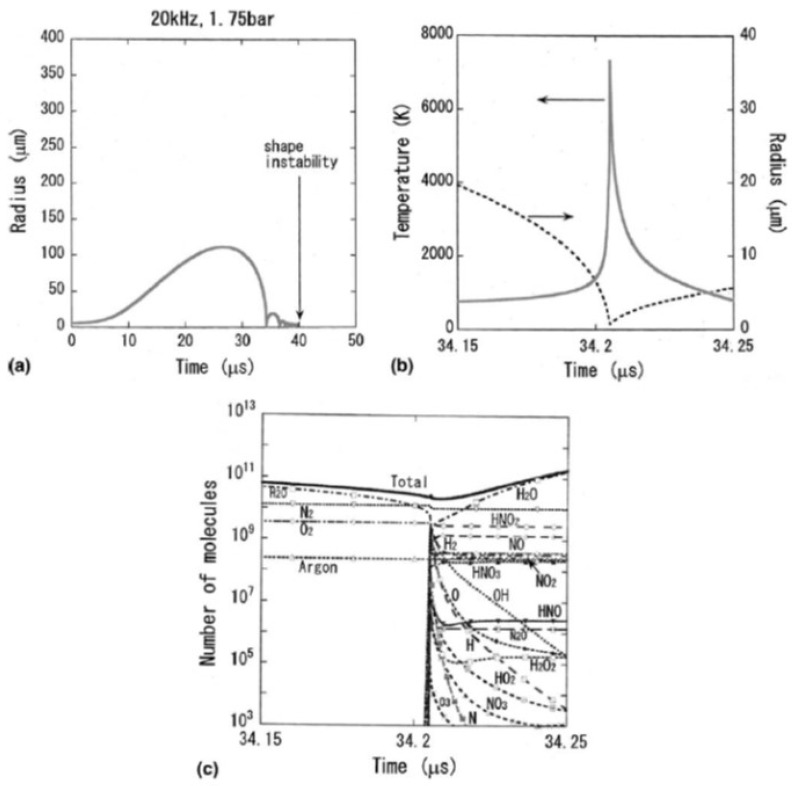
Calculated results when the acoustic amplitude is 1.75 bar [33]. The other conditions are the same as those in Figure 8. (**a**) The radius-time curve for one acoustic cycle. A bubble disintegrates into daughter bubbles by shape instability at t = 39.85 μs. (**b**) The temperature inside a bubble and the bubble radius as a function of time at around the end of the bubble collapse only for 0.1 μs. (**c**) Numbers of molecules of various species inside a bubble as a function of time with the same time axis as that of (**b**). The vertical axis is in logarithmic scale. Copyright 2005, with the permission from Elsevier.

Gaseous bubbles appear at relatively low acoustic amplitudes at relatively low ultrasonic frequencies (Figure 9) or at relatively high ultrasonic frequencies [33,35]. Under the conditions, the bubble expansion is not so intense, and correspondingly, evaporation of water vapor into a bubble is not so intense. Thus, intense condensation of water vapor at the bubble collapse results in the main bubble content being noncondensable gas such as air at the end of the bubble collapse (Figure 9c) [33]. As the amount of water vapor inside a gaseous bubble is much smaller than that in a vaporous bubble, the peak bubble temperature (7300 K) inside a gaseous bubble is higher than that (6300 K) inside a vaporous bubble because water vapor considerably cools a bubble by endothermic heat of vapor dissociation.

**Figure 10:**
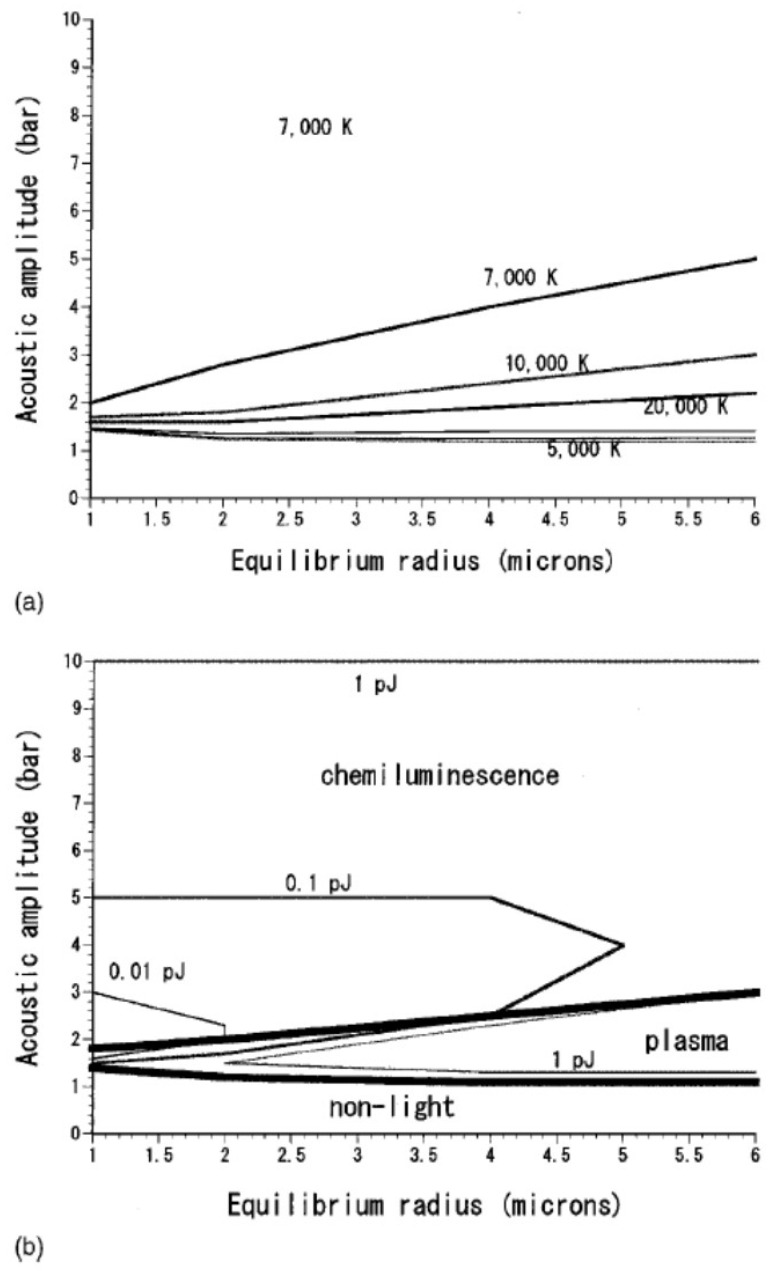
Results of numerical simulations of bubble pulsations for various acoustic amplitudes and equilibrium bubble radii for an argon bubble in 5 °C water irradiated by 20 kHz ultrasound [34]. (**a**) The bubble temperature at the collapse. The isothermal lines are from the bottom 5000, 10,000, 20,000, 20,000, 10,000, and 7000 K. Above the isothermal line of 7000 K, the bubble temperature at the collapse is independent of acoustic amplitude and is always 7000 K. (**b**) The mechanism of the light emission. At large acoustic amplitudes chemiluminescence is relatively strong; OH* → OH + hν, where OH* is created by the reactions O + H + M → OH* + M and OH + H + OH → OH* + H_2_O where M is the third body. At lower acoustic amplitudes emissions from plasma are dominant; electron bremsstrahlung and radiative recombination of electrons and ions. At very low acoustic amplitudes no light is emitted. The energy of the emitted light per bubble collapse is also shown. Copyright 2001, with the permission of AIP Publishing.

The results of numerical simulations for an argon bubble in 5 °C water irradiated by 20 kHz ultrasound are summarized in Figure 10 [34]. As already discussed, the peak bubble temperature is nearly constant at relatively high acoustic amplitudes (Figure 10a). As the liquid temperature is lower than that in Figure 8, the peak bubble temperature of vaporous bubbles in Figure 10 is higher than that in Figure 8 [25].

According to the numerical simulations, the mechanism of the SL light emission from vaporous bubbles is chemiluminescence of OH as well as emissions from weakly ionized plasma formed inside a bubble (Figure 10b) [34]. The intensity of chemiluminescence of OH is estimated by the rates of the reactions O + H + M → OH* + M and OH + H + OH → OH* + H_2_O, where OH* is the excited OH radical and M is the third body. The excited OH radical emits light (chemiluminescence) by OH* → OH + hν. The quenching of molecular emissions by collisional deexcitation is taken into account in the calculations [34]. Inside a bubble at the end of the bubble collapse, temperature and pressure are extremely high, which results in strong quenching of molecular emissions by collisional deexcitation. Nevertheless, chemiluminescence of OH is still intense in vaporous bubbles.

Ndiaye et al. [37] experimentally reported that the relative vibrational population distribution of OH obtained from the MBSL spectra deviates strongly from the equilibrium Boltzmann distribution. This may be caused by the chemiluminescence origin of OH-line emissions.

For gaseous argon bubbles, the peak temperature is as high as 20,000 K (Figure 10a) [34]. At such high bubble temperature, considerable thermal ionization occurs inside a bubble. Furthermore, ionization potential of gas molecules is considerably lowered by the extreme high density inside a bubble [25]. The density inside a bubble is nearly as high as that of condensed phase. Pressure inside a bubble at the end of the bubble collapse is as high as about 10 GPa [23]. There is some experimental evidence on the considerable ionization-potential lowering in SL bubbles [38]. The ionization potential lowering is due to overlaps of electron wavefunctions in the extremely dense gas inside a bubble [25,39,40]. It is one of the main reasons for the plasma formation inside a bubble in addition to the high bubble temperature at the end of the violent bubble collapse. With regard to the degree of ionization potential lowering, further studies are required.

The degree of ionization inside a gaseous bubble is known to be 0.03~300% with ionization potential lowering by 40~75% or more [38,41,42,43]. It results in the dominant light emissions from plasma such as electron-atom and electron-ion bremsstrahlung, radiative recombination of electrons and ions, radiative attachment of electrons to neutral atoms, etc. (Figure 10b) [25,34,43].

**Figure 11:**
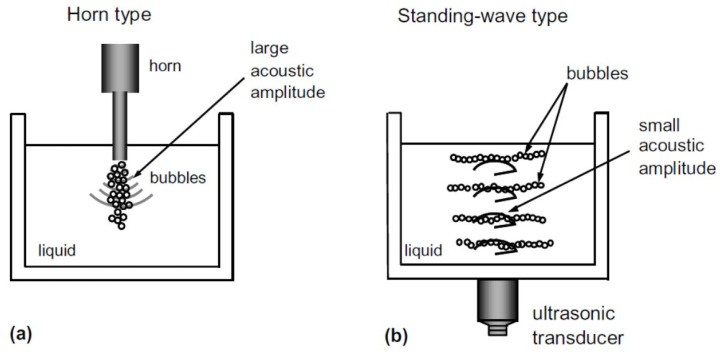
Two types of sonochemical reactors [33]. (**a**) A horn-type reactor. (**b**) A standing-wave type reactor. Copyright 2005, with the permission from Elsevier.

There are mainly two types in the methods of ultrasonic irradiation of liquid (Figure 11) [33]. One is the method to use an ultrasonic horn immersed in the liquid (Figure 11a). The other is the method of ultrasonic bath type (standing-wave type) (Figure 11b). In the horn-type, acoustic amplitude near the horn tip can be very high such as 10 bar. On the other hand, in the standing-wave type, bubbles are repelled from the pressure antinodes when the acoustic amplitude is higher than about 1.77 bar at 20 kHz due to the acoustic radiation force acting on bubbles called primary Bjerknes force [5,33,44]. In other words, many actively pulsating bubbles gather at the region where acoustic amplitude is about 1.77 bar at 20 kHz [45].

A SBSL bubble is a gaseous bubble because acoustic amplitude in SBSL is much lower than the threshold one for repulsion of the primary Bjerknes force [6].

**Figure 12:**
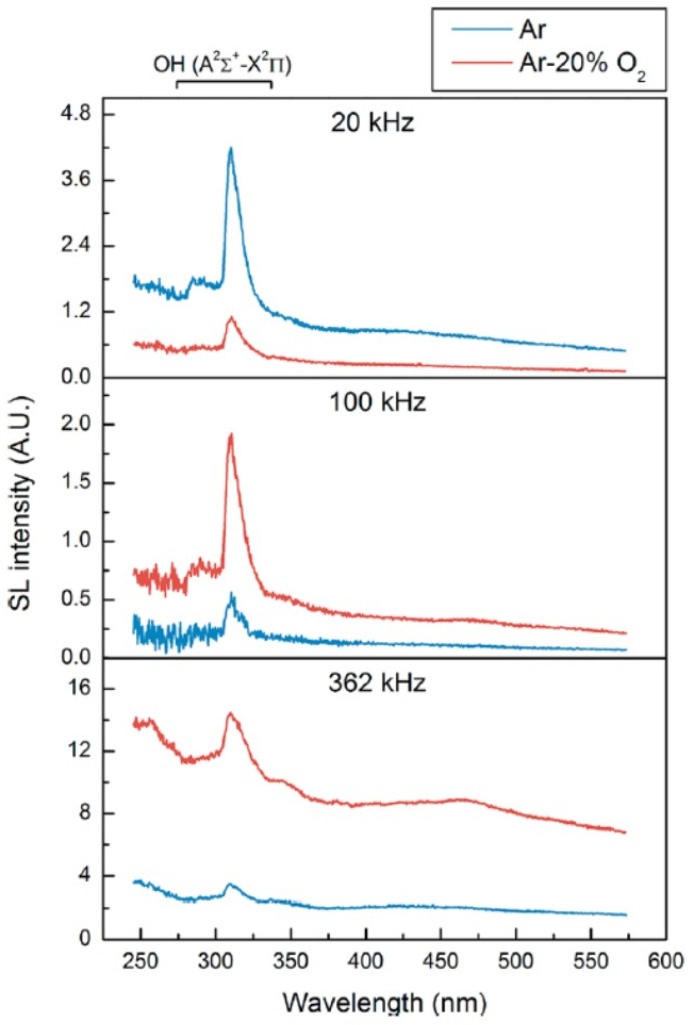
MBSL spectra of water at 20 kHz with 33 W, 100 kHz with 40 W, and 362 kHz with 43 W at 18 °C [46]. The apparent broad peak around 400–475 nm at 362 kHz is an artifact due to second-order light emission of the strong MBSL UV part. Copyright 2018, with the permission from American Chemical Society.

Considering the above discussions, many bubbles in the horn-type reactors are vaporous bubbles. On the other hand, many bubbles in the standing-wave type reactors are gaseous bubbles. Thus, it is suggested that SL emission in the horn-type is mainly by chemiluminescence of OH and emissions from weakly ionized plasma formed inside a bubble, and that in the standing-wave type it is mainly by the emissions from plasma. Furthermore, more gaseous bubbles are present at higher ultrasonic frequencies due to less expansion of a bubble caused by shorter acoustic period, as discussed later [35]. Thus, at higher ultrasonic frequencies, emissions from plasma are much more stronger than chemiluminescence of OH compared to MBSL at lower ultrasonic frequencies. It has been actually observed experimentally (Figure 12 [46]). The emissions from plasma, which is the continuum component of the MBSL spectra, is stronger at higher ultrasonic frequencies relative to the intensity of OH-line at the wavelength of 310 nm.

**Figure 13:**
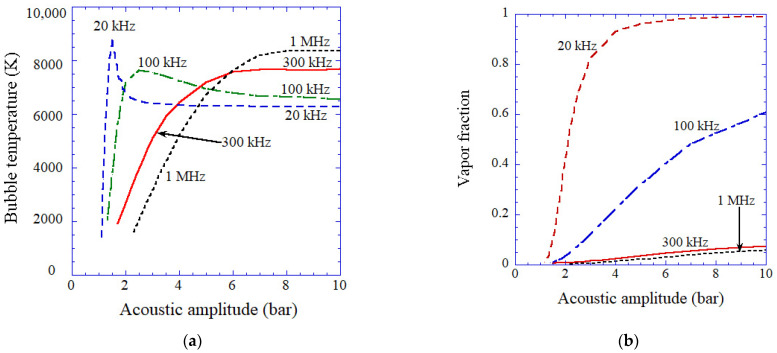
The calculated results as a function of acoustic amplitude for various ultrasonic frequencies (20 kHz, 100 kHz, 300 kHz, and 1 MHz) for the first collapse of an isolated spherical air bubble [35]. The ambient bubble radii are 5 μm for 20 kHz, 3.5 μm for 100 and 300 kHz, and 1 μm for 1 MHz. (**a**) The temperature inside a bubble at the bubble collapse. (**b**) The molar fraction of water vapor inside a bubble at the end of the bubble collapse. Copyright 2007, with the permission of AIP Publishing.

As already discussed, at relatively low ultrasonic frequencies, vaporous bubbles are seen at high acoustic amplitudes (Figure 13) [35]. The vapor fraction inside a bubble at the end of the bubble collapse increases as acoustic amplitude increases (Figure 13b). For 20 kHz and 100 kHz in Figure 13, the bubble temperature is the highest at a relatively low acoustic amplitude because at higher acoustic amplitude vapor fraction becomes higher. For 300 kHz and 1 MHz in Figure 13, on the other hand, vapor fraction is always less than 0.1, and gaseous bubbles are seen even at relatively high acoustic amplitudes. Thus, the bubble temperature increases as the acoustic amplitude increases and becomes nearly constant at relatively high acoustic amplitudes (Figure 13a).

## 4. Influence of Bubble Size

**Figure 14:**
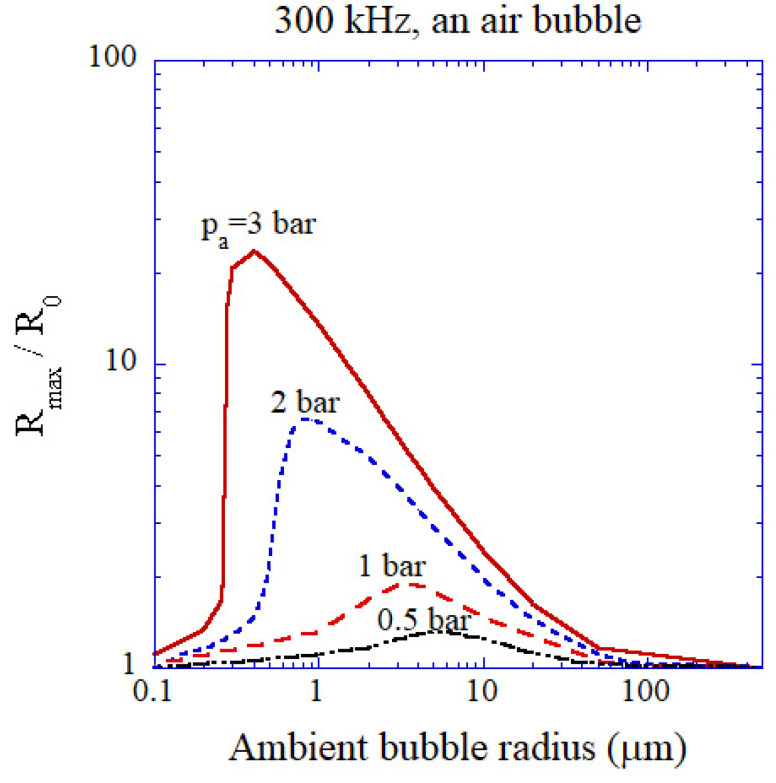
The calculated expansion ratio (R_max_/R_0_) as a function of ambient bubble radius for various acoustic amplitudes at 300 kHz [43]. Both the horizontal and vertical axes are in logarithmic scale. Copyright 2008, with the permission of AIP Publishing.

The linear resonance radius of an air bubble is 10.9 μm at 300 kHz [5,43]. However, due to the strong nonlinear nature of bubble pulsation, the expansion ratio (R_max_/R_0_), where R_max_ is the maximum bubble radius and R_0_ is the ambient bubble radius which is the bubble radius when ultrasound is absent, takes the peak value at smaller ambient bubble radius than the linear resonance radius even at acoustic amplitude as low as 0.5 bar (Figure 14) [43]. As the acoustic amplitude increases, the maximal response of a bubble shifts toward smaller ambient radius. At the acoustic amplitude of 3 bar, the range of ambient bubble radius for higher expansion ratio than 3 is from 0.27 to 7 μm [43].

**Figure 15:**
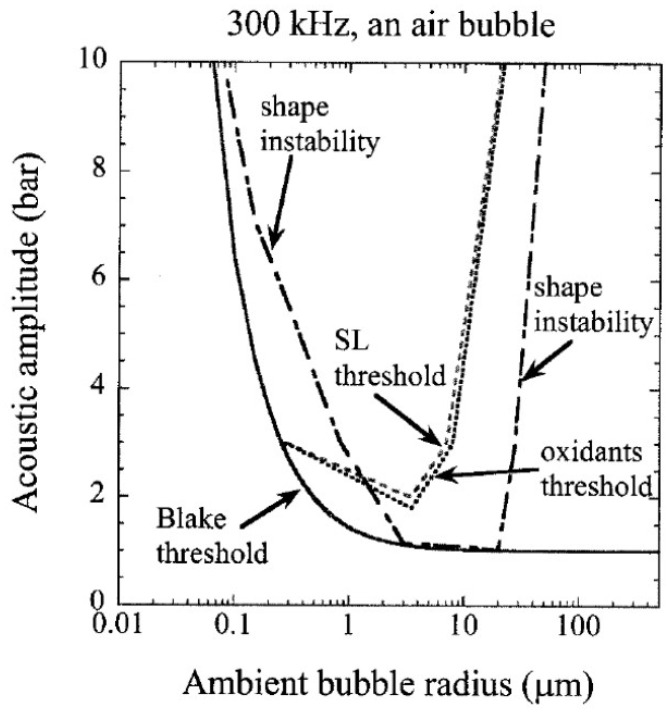
The calculated thresholds for the shape instability, for SL, and for oxidant production as well as the Blake threshold in R_0_-p_a_ plane [43]. Copyright 2008, with the permission of AIP Publishing.

The shape instability of a bubble is another important factor to determine the range of ambient bubble radius for active bubbles observed experimentally [43]. When the acoustic amplitude is above the threshold for shape instability shown in Figure 15 by the dash-dotted line, a bubble disintegrates into daughter bubbles in a few acoustic cycles [43]. The method for the numerical calculations of the threshold for shape instability is described in Refs. [35,47,48]. When the acoustic amplitude is larger than the Blake threshold shown by a solid line in Figure 15, bubble expansion becomes very strong, which is called transient cavitation [5,43]. When the threshold SL intensity and that for sonochemical production of oxidants are assumed as 10^−7^ pJ/collapse and 10^8^ molecules/s, respectively, they are equal to or higher than the Blake-threshold acoustic amplitude as shown in Figure 15 [43]. The typical range of ambient radius for actual active bubbles is from the SL threshold radius (or threshold for sonochemical production of oxidants) to slightly above the threshold radius for shape instability because larger active bubbles disintegrate into daughter bubbles in a few acoustic cycles. Indeed, Brotchie et al. [49] reported experimentally that the range of ambient radius for MBSL bubbles was from 2.9 to 3.5 μm at 355 kHz, which is a relatively narrow range near the threshold for shape instability. Further studies are required on this topic [18,50].

**Figure 16:**
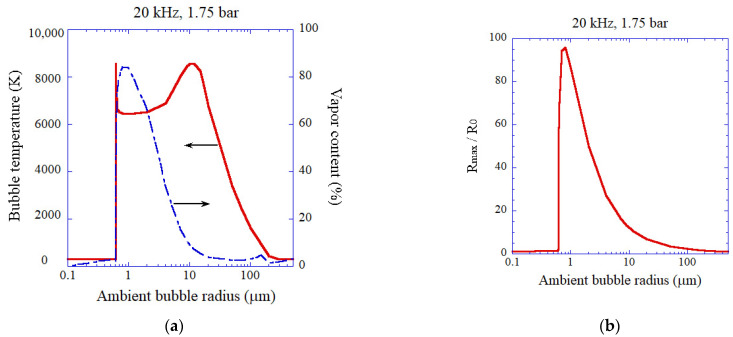

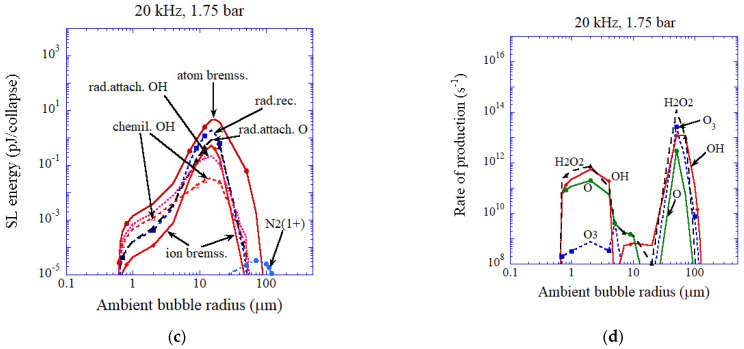
The calculated results for an air bubble as a function of ambient bubble radius at 20 kHz and 1.75 bar in ultrasonic frequency and acoustic amplitude, respectively [43]. The horizontal axis is in logarithmic scale. (**a**) The peak temperature (solid) and the molar fraction of water vapor (dash dotted) inside a bubble at the end of the bubble collapse. (**b**) The expansion ratio (R_max_/R_0_). (**c**) The intensity of each emission process in SL. The vertical axis is in logarithmic scale. (**d**) The rate of production of each oxidant with the logarithmic vertical axis. Copyright 2008, with the permission of AIP Publishing.

The dependence of peak bubble temperature as well as vapor fraction inside an air bubble at the end of the bubble collapse on ambient bubble radius is shown in Figure 16a for ultrasound of 20 kHz and 1.75 bar [43]. The bubble temperature is the highest for relatively low vapor fraction (gaseous bubbles) as already discussed in the previous section. The vapor fraction is correlated with the expansion ratio of a bubble shown in Figure 16b. For vaporous bubbles, the bubble temperature is around 6500 K.

The dependence of the light intensity of each emission process in MBSL on ambient bubble radius is shown in Figure 16c for ultrasound of 20 kHz and 1.75 bar [43]. The main emission process is electron-atom bremsstrahlung. The MBSL intensity strongly depends on the ambient bubble radius through the peak bubble temperature and the emission volume. The vertical axis of Figure 16c is in logarithmic scale. For vaporous bubbles, chemiluminescence of OH as well as electron-atom bremsstrahlung and radiative attachment of electrons to OH molecules are important emission processes. For gaseous bubbles, on the other hand, electron-atom bremsstrahlung and radiative recombination of electrons and ions are much more intense than chemiluminescence of OH as already discussed in the previous section.

The rate of production of each oxidant inside an air bubble is shown as a function of ambient bubble radius in Figure 16d with the logarithmic vertical axis [43]. It is seen that at relatively high bubble temperatures the amounts of oxidants produced become relatively small. It is because at high bubble temperatures oxidants are strongly consumed inside a bubble by oxidizing nitrogen [22,43,51]. As a result, the amount of HNO_2_, NO, and NO_2_ produced inside an air bubble is relatively large under the condition.

**Figure 17:**
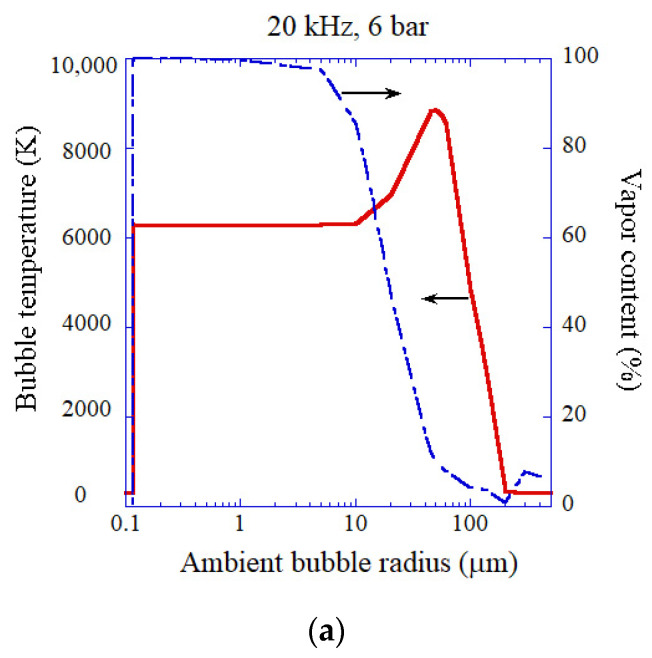

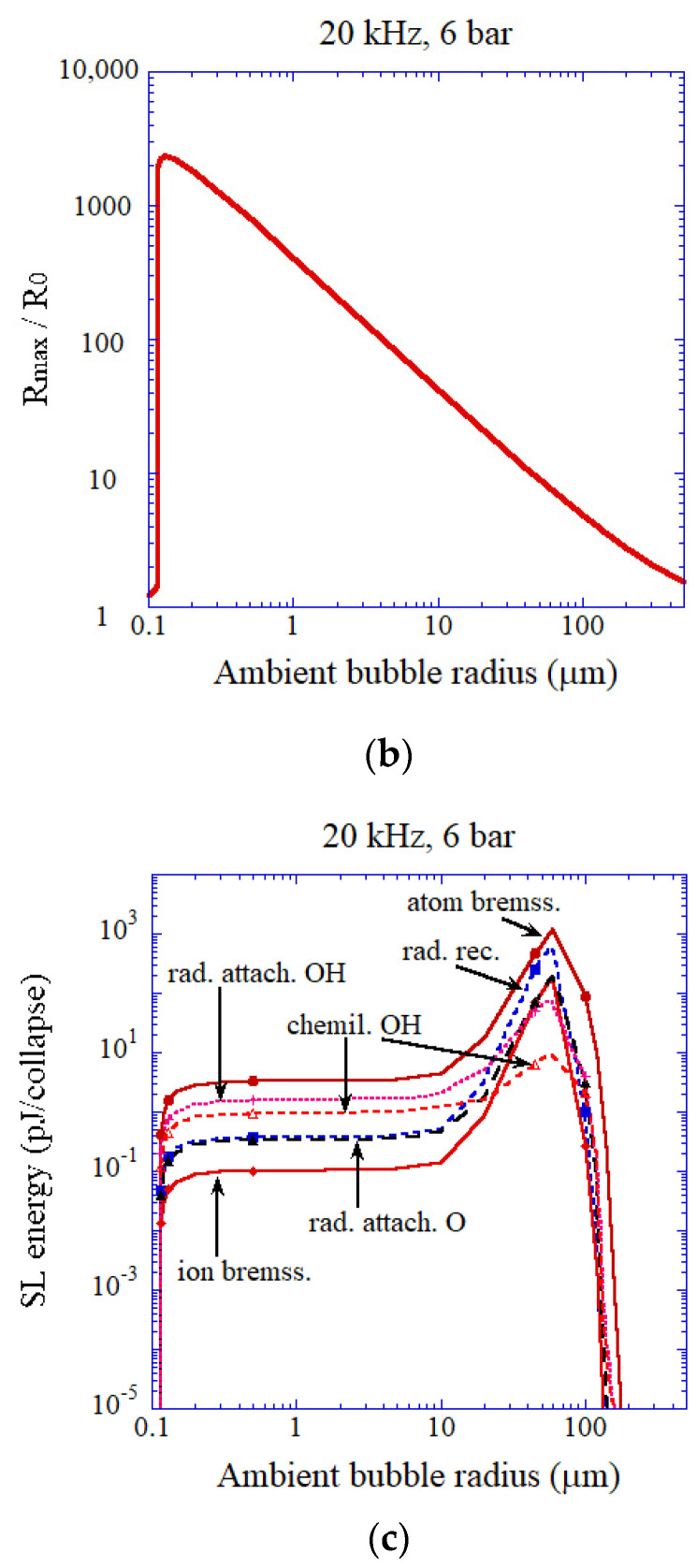
The calculated results for an air bubble as a function of ambient bubble radius at 20 kHz and 6 bar in ultrasonic frequency and acoustic amplitude, respectively [43]. The horizontal axis is in logarithmic scale. (**a**) The peak temperature (solid) and the molar fraction of water vapor (dash dotted) inside a bubble at the end of the bubble collapse. (**b**) The expansion ratio (R_max_/R_0_). (**c**) The intensity of each emission process in SL. The vertical axis is in logarithmic scale. Copyright 2008, with the permission of AIP Publishing.

The calculated results for acoustic amplitude of 6 bar at 20 kHz for an air bubble are shown as a function of ambient bubble radius in Figure 17 [43]. When the ambient radius is larger than 0.12 μm, which is slightly larger than the Blake threshold radius (0.11 μm), and smaller than about 10 μm, bubbles are vaporous ones with nearly constant peak temperature of about 6300 K (Figure 17a). The maximum peak temperature is 8700 K at the ambient bubble radius of 50 μm due to relatively small vapor fraction (gaseous bubble). The expansion ratio (R_max_/R_0_) takes the maximum value of about 2400 at the ambient radius of 0.13 μm, which is three orders of magnitude smaller than the linear resonance radius of 164 μm at 20 kHz due to strong nonlinearity of the bubble pulsation (Figure 17b) [43].

As in the case of Figure 16c, the main light emission process in MBSL is electron-atom bremsstrahlung. For vaporous bubbles, chemiluminescence of OH is another important emission process. For gaseous bubbles, emissions from plasma (electron-atom bremsstrahlung and radiative recombination of electrons and ions) are about two orders of magnitude stronger in intensity than chemiluminescence of OH as already discussed. The maximum SL intensity is $2.0\times 10^{3}$ pJ per bubble collapse at the ambient radius of 60 μm. Under this condition, the degree of ionization inside an air bubble is 0.5% with the ionization potential lowering of about 50% [43].

## 5. Bubble–Bubble Interaction

In contrast to the case of the single bubble system (SBSL), some complexity arises from the bubble–bubble interaction in multibubble system (MBSL). In a bubble cloud, bubble pulsation is strongly influenced by the pulsations of surrounding bubbles because they radiate acoustic waves into the liquid, which is called the bubble–bubble interaction [5].

The acoustic pressure (p) radiated from a pulsating bubble is given as follows [5].

(4) $$p=\frac{\rho_{L,\infty }}{r}\left(2R{\dot{R}}^{2}+R^{2}\ddot{R}\right)$$

where r is the distance from a radiating bubble. Accordingly, the Keller equation (Equation (1)) is approximately modified as follows taking into account the bubble–bubble interaction [5,19].

(5) $$\begin{array}{l} \left(1-\frac{\dot{R}}{c_{\infty }}\right)R\ddot{R}+\frac{3}{2}{\dot{R}}^{2}\left(1-\frac{\dot{R}}{3c_{\infty }}\right)=\frac{1}{\rho_{L,\infty }}\left(1+\frac{\dot{R}}{c_{\infty }}\right)\left[p_{B}-p_{s}\left(t\right)-p_{\infty }\right]+\frac{R}{c_{\infty }\rho_{L,\infty }}\frac{dp_{B}}{dt}-\\ \sum_{i}\frac{1}{r_{i}}\left(2R_{i}{\dot{R}}_{i}^{2}+R_{i}^{2}{\ddot{R}}_{i}\right) \end{array}$$

where $r_{i}$ is the distance from the bubble numbered *i*, $R_{i}$ is the instantaneous radius of the bubble numbered *i*, and summation is for all the surrounding bubbles. When the radius of each bubble is assumed to be the same for all the bubbles, Equation (5) is simplified as follows [5,19,52,53,54].

(6) $$\begin{array}{l} \left(1-\frac{\dot{R}}{c_{\infty }}\right)R\ddot{R}+\frac{3}{2}{\dot{R}}^{2}\left(1-\frac{\dot{R}}{3c_{\infty }}\right)=\frac{1}{\rho_{L,\infty }}\left(1+\frac{\dot{R}}{c_{\infty }}\right)\left[p_{B}-p_{s}\left(t\right)-p_{\infty }\right]+\frac{R}{c_{\infty }\rho_{L,\infty }}\frac{dp_{B}}{dt}-\\ S\left(2R{\dot{R}}^{2}+R^{2}\ddot{R}\right) \end{array}$$

where S is the coupling strength of the bubble–bubble interaction and given as follows when the spatial distribution of bubbles is assumed to be uniform.

(7) $$S=\sum_{i}\frac{1}{r_{i}}=\int_{l_{min}}^{l_{max}}\frac{4\pi r^{2}n}{r}dr=2\pi n\left(l_{max}^{2}-l_{min}^{2}\right)\approx 2\pi nl_{max}^{2}$$

where $l_{max}$ is the radius of a bubble cloud, $l_{min}$ is the distance between a bubble and a nearest bubble, $l_{max}\gg l_{min}$ is assumed in the last equation, and n is the number density of bubbles.

**Figure 18:**
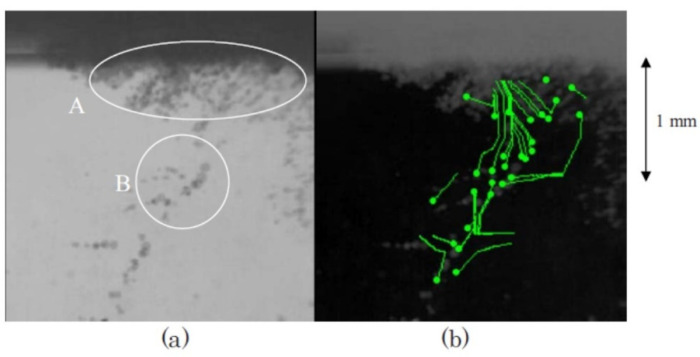
Cavitation bubbles under an ultrasonic horn at 29 kHz and 5 W observed by a high-speed video camera [19]. The frame (**b**) is the result of the analysis of the bubble motion. The small circles are the starting points for the analysis and the curves are the calculated streamlines of bubbles. In the frame (**a**), the bubble clouds A and B are marked by circles. Copyright 2008, with the permission from American Physical Society.

**Figure 19:**
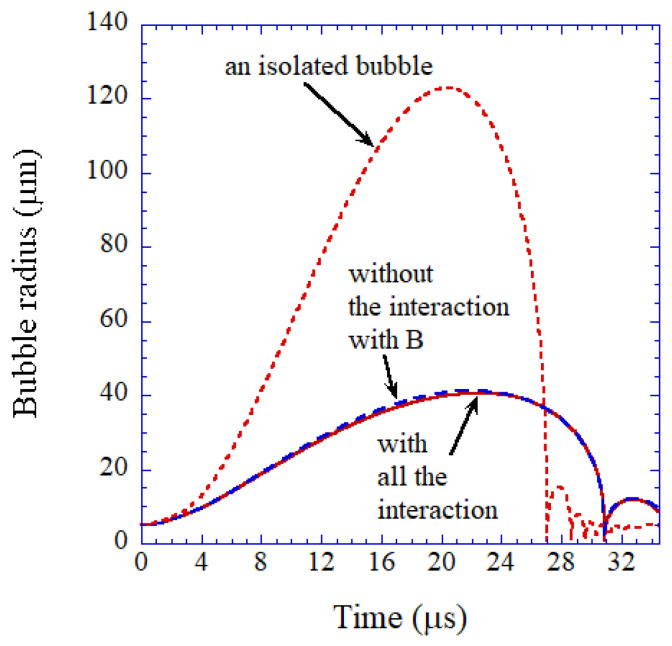
Calculated radius of a bubble in the cloud A in Figure 18 as a function of time for one acoustic cycle at 29 kHz and 2.36 bar in frequency and pressure amplitude of ultrasound, respectively [19]. The ambient bubble radius is 5 μm. The dotted line is the calculated result neglecting all the interactions with other bubbles (an isolated bubble). The dashed line is the calculated result neglecting only the interaction with the bubbles in the cloud B. The solid line is the calculated result taking into account all the interactions with surrounding bubbles. Copyright 2008, with the permission from American Physical Society.

Cavitation bubbles under an ultrasonic horn shown in Figure 18 have been numerically analyzed using Equation (6) as shown in Figure 19 [19]. Due to the bubble–bubble interaction, bubble expansion is strongly suppressed [5,19,53].

According to the high-speed video image shown in Figure 18, many bubbles move upward toward the horn tip [19]. There are three kinds of forces acting on a bubble in an acoustic field [5,19]. One is the radiation force from an acoustic wave (ultrasound) called the primary Bjerknes force. Another is the force acting from other bubbles called the secondary Bjerkes force. The other is the buoyant force. Numerical evaluation of the three kinds of forces on a bubble has indicated that bubbles in the bubble cloud B in Figure 18 move upward toward the horn tip by the secondary Bjerknes force from bubbles in the bubble cloud A [19].

**Figure 20:**
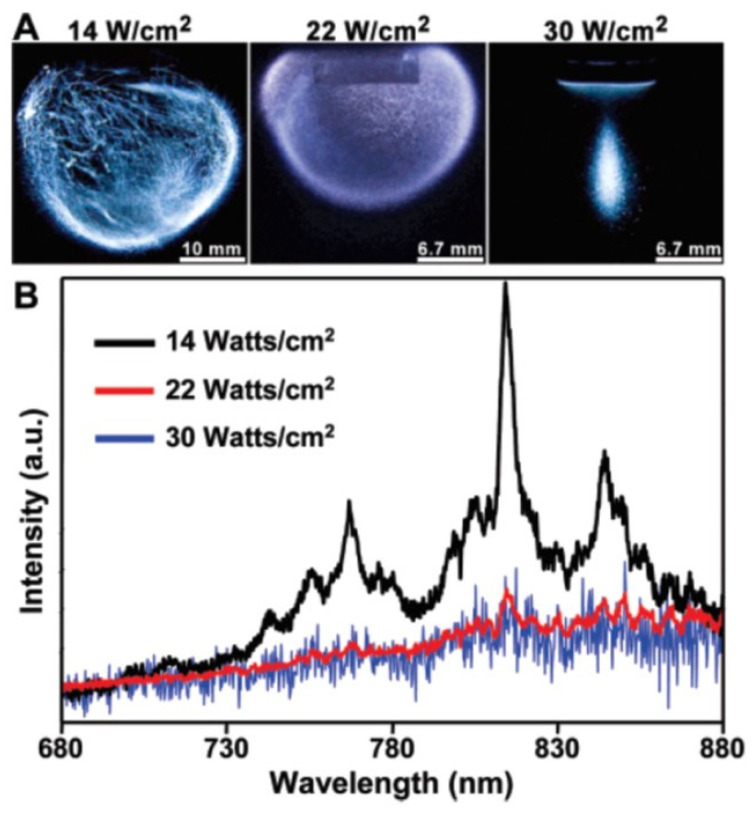
MBSL of concentrated H_2_SO_4_ at different acoustic powers of an ultrasonic horn [55]. (**A**) Photographs (10 s exposures) of different light emitting regimes of MBSL of H_2_SO_4_, from left to right, with increasing acoustic intensity, filamentous, bulbous, and cone shaped emission; (**B**) MBSL spectra of concentrated H_2_SO_4_ at the three acoustic intensities shown. As the acoustic power is increased, the Ar lines become weaker. Copyright 2007, with the permission from American Chemical Society.

MBSL intensity in sulfuric acid is much greater than that in water [2,55,56,57]. One of the main reasons for the much brighter MBSL in sulfuric acid is much lower saturated vapor pressure than that of water because it results in higher bubble temperature. According to Eddingsaas and Suslick [55], large and abrupt changes in bubble–cloud dynamics, MBSL light intensity, and its spectra under an ultrasonic horn were experimentally observed upon varying the acoustic power in concentrated (95 wt %) sulfuric acid saturated with Ar (Figure 20). There were three different MBSL regimes as a function of acoustic intensity [55]. At relatively low acoustic intensities, a wispy, filamentous MBSL with strong Ar and SO lines was observed. Above about 16 W/cm^2^, the cavitating bubbles suddenly formed a bulb near the horn tip, creating a small globe of MBSL with very weak Ar lines along with the broad continuum. Above about 24 W/cm^2^, MBSL was observed from a cone at the horn tip, and the spectra consisted only of the broad continuum without Ar lines. These behaviors may be related to stronger bubble–bubble interaction as well as stronger secondary Bjerknes force at higher acoustic power mainly due to higher number density of bubbles. An [58] has already numerically studied the influence of the bubble–bubble interaction on MBSL spectra for Ar bubbles in water. Further studies are required on this topic.

## 6. Acoustic Field

**Figure 21:**
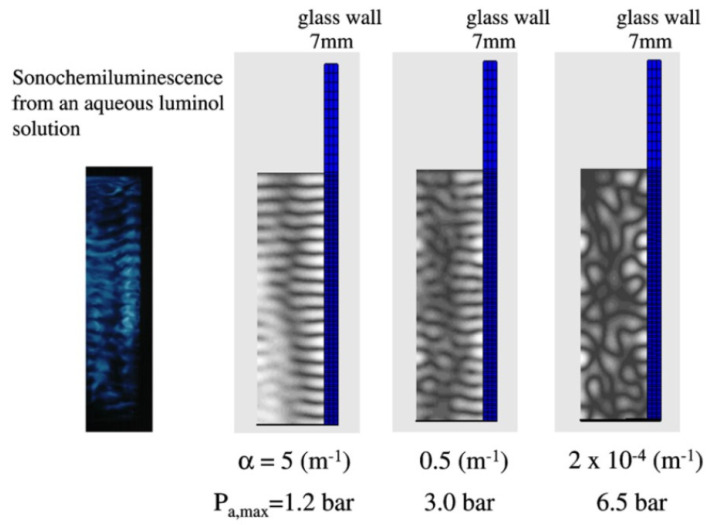
The calculated spatial distribution of the acoustic amplitude for glass wall (7 mm in thickness) for various attenuation coefficients of ultrasound [66]. The full-width at half maximum for the Gaussian distribution of the vibration amplitude of the vibrating plate at the bottom is 5 cm. The wall height is 20 cm. A half of the width (the full width is 7 cm) of the liquid container is shown. The photograph of sonochemiluminescence from an aqueous luminol solution is also shown for the corresponding half plane. Copyright 2007, with the permission from Elsevier.

MBSL has been used to visualize acoustic fields through the spatial distribution of active bubbles in SL [59,60,61,62]. Sonochemiluminescence (SCL) has also been used to visualize acoustic fields, where SCL is the light emission from an aqueous luminol solution by chemical reactions of luminol with oxidants such as OH radicals and H_2_O_2_ produced from cavitation bubbles [62,63,64,65]. An example of the SCL image is shown for a half plane of a rectangular cell (5 cm × 5 cm × 14.5 cm) at 140 kHz in Figure 21 [62,66]. Horizontal stripes of pressure nodes and antinodes are seen in the photograph, where pressure antinodes are the bright regions in blue by SCL. In addition, a vertical narrow dark region is seen in the photograph of Figure 21, where acoustic amplitude is relatively low (or number density of bubbles is relatively low).

In Figure 21, the numerically calculated spatial distributions of acoustic fields by the finite element method (FEM) are also shown for comparison for various attenuation coefficients (α) [66]. In the FEM program, the acoustic field in a rectangular cell filled with water with a vibrating plate at the bottom is coupled with the vibration of the side wall of the cell. The vibration amplitude at the center of the vibrating plate is assumed as 0.1 μm. The calculated result with the attenuation coefficient of 2 × 10^−4^ m^−1^ corresponds to the case that there is no bubble in the liquid. In this case, strong vibration of the side wall by high acoustic amplitude influences the acoustic field in the cell significantly because the side wall strongly radiates acoustic waves into the liquid. As a result, no horizontal stripes of pressure antinodes and nodes are seen.

For the attenuation coefficient of 5 m^−1^, the horizontal stripes of pressure antinodes and nodes are seen as well as a vertical narrow dark region, which is qualitatively similar to the spatial distribution of SCL as compared in Figure 21 [66]. For the attenuation coefficient of 0.5 m^−1^, some stripes of pressure antinodes are disconnected. Thus, the actual attenuation coefficient in the experiment of SCL is probably about 5 m^−1^.

**Figure 22:**
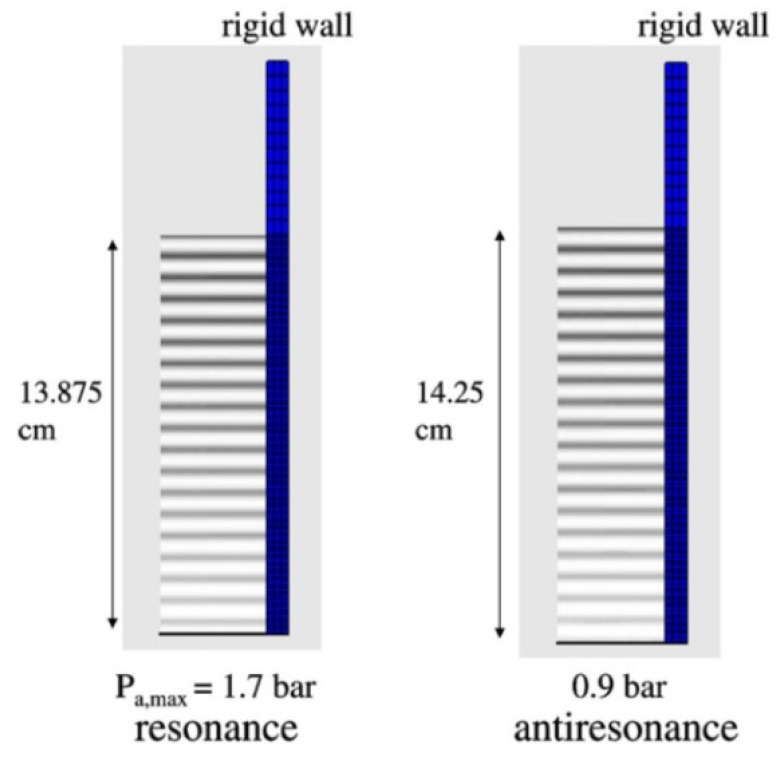
The calculated spatial distribution of the acoustic amplitude for the resonance liquid height (13.875 cm) and the antiresonance one (14.25 cm) for the rigid wall [66]. The bottom plate vibrates spatially uniformly. The attenuation coefficient is 5 m^−1^. Copyright 2007, with the permission from Elsevier.

For rigid side wall with spatially uniform vibration amplitude of the vibrating plate at the bottom of the cell, the horizontal stripes of pressure antinodes and nodes are parallel as shown in Figure 22 [66]. When the vibration frequency of the vibrating plate is 100 kHz, the liquid height of 13.875 cm and 14.25 cm correspond to resonance and antiresonance, respectively. For the both cases, the liquid surface is pressure node. For the resonance case, the surface of the vibrating plate coincides with pressure antinode, while it coincides with pressure node for the antiresonance case.

**Figure 23:**
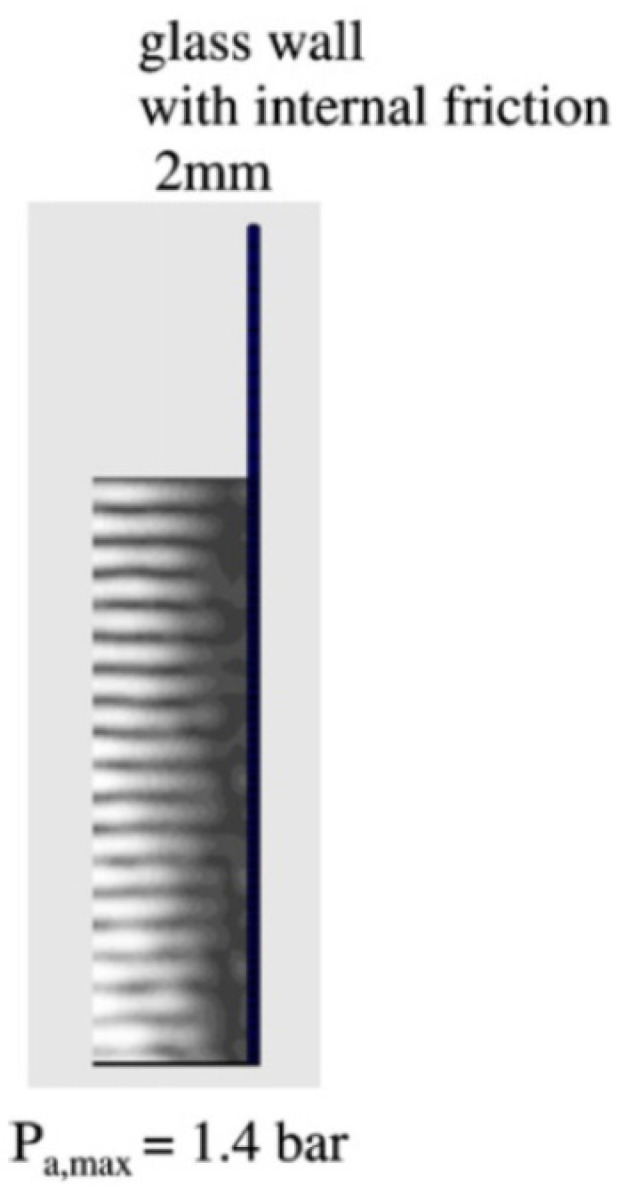
The calculated spatial distribution of the acoustic amplitude for glass wall of 2 mm in thickness [66]. The attenuation coefficient is 5 m^−1^. Copyright 2007, with the permission from Elsevier.

When the glass side wall is thin (2 mm thick), the surface of the thin side wall nearly coincides with vertical pressure node as seen in Figure 23 because it is nearly a free surface [66].

**Figure 24:**
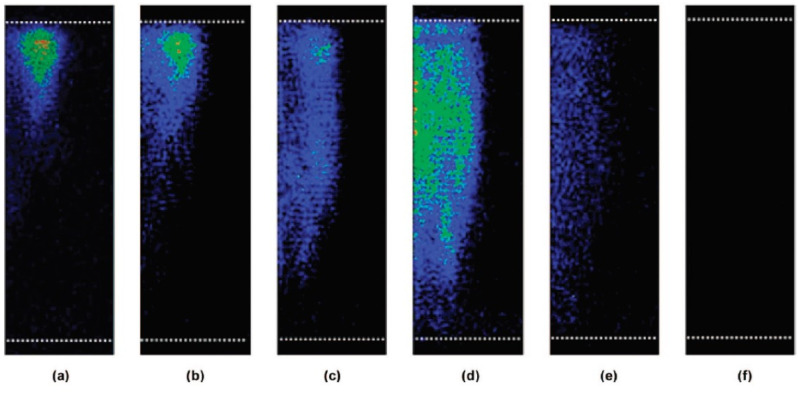
Images showing the effect of dissolved oxygen concentration on the MBSL structure taken from the side of the vessel: (**a**) 8.5, (**b**) 7.0, (**c**) 5.6, (**d**) 4.2, (**e**) 3.4, and (**f**) 2.9 mg/L [59]. The white dotted lines above and below the MBSL structure denotes the liquid surface and the transducer position, respectively. Continuous sonication at a frequency of 448 kHz and a power of 1.1 W/cm^2^ was applied. Exposure time was set to collect 2 × 10^5^ acoustic cycles. The center axis of the vessel is located on the left side of the images. Copyright 2008, with the permission from American Chemical Society.

Spatial distribution of MBSL intensity strongly depends on dissolved air concentration in water as shown in Figure 24 [59]. In Figure 24, the dissolved oxygen concentration is shown as an indicator of dissolved air concentration. For air saturated water irradiated with ultrasound of 448 kHz and 1.1 W/cm^2^, the spatial distribution of MBSL intensity is localized near the liquid surface as shown in Figure 24a. At the oxygen concentration of 4.2 mg/L, which corresponds to about 50% in degree of air saturation, the spatial distribution of MBSL intensity is nearly uniform inside the liquid as shown in Figure 24d. For even lower oxygen concentrations, MBSL intensity gradually diminishes.

What is the reason for the dependence of the spatial distribution of MBSL on dissolved air concentration? For air saturated water, there are many visible large bubbles in the liquid under ultrasound. For the degree of air saturation of about 50%, there are very few visible large bubbles in the liquid. It suggests that visible large bubbles cause Anderson localization of an acoustic wave (ultrasonic wave) and that consequently spatial distribution of acoustic amplitude becomes strongly inhomogeneous [67]. It may result in the spatially inhomogeneous distribution of MBSL intensity in the presence of visible large bubbles. Further studies are required on this topic.

**Figure 25:**
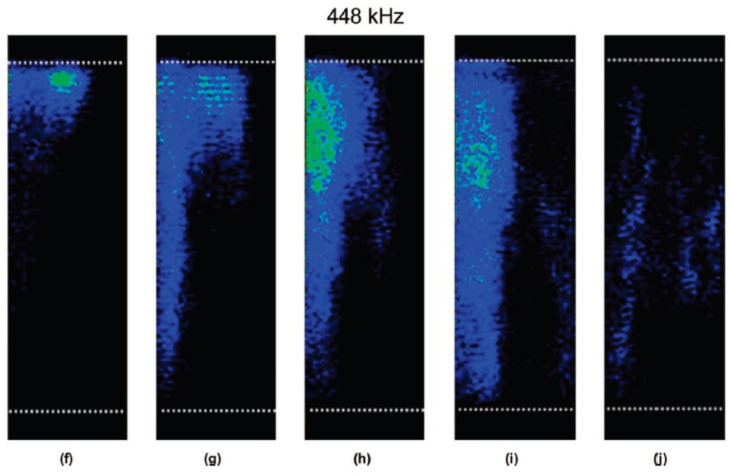
Images showing the effect of increasing pulse-off time on the MBSL structure generated in saturated water at a frequency of 448 kHz and at a power of 1.1 W/cm^2^: (**f**) 1.5, (**g**) 10, (**h**) 40, (**i**) 110, and (**j**) 135 ms [59]. The pulse-on time was fixed at 5 ms. The white dotted lines above and below the MBSL structure denotes the liquid surface and the transducer position, respectively. The center axis of the vessel is located on the left side of the images. Copyright 2008, with the permission from American Chemical Society.

Under the pulsed ultrasound irradiation, spatial distribution of MBSL intensity strongly depends on pulse-off time under a fixed pulse-on time as shown in Figure 25 [59]. For a very short pulse-off time, the spatial distribution of MBSL intensity is localized near the liquid surface as shown in Figure 25f. For the pulse-off time of 110 ms under fixed pulse-on time of 5 ms at 448 kHz, the spatial distribution of MBSL intensity is nearly homogeneous as shown in Figure 25i. Further increase in pulse-off time results in diminishing of MBSL intensity as shown in Figure 25j. As in the case of dissolved air concentration, this behavior is related to the presence and absence of visible large bubbles in the liquid. For a very short pulse-off time of 1.5 ms in Figure 25f, there are many visible large bubbles in the liquid. On the other hand, for the pulse-off time of 110 ms, there are very few visible large bubbles in the liquid. It may result in the less Anderson localization of an acoustic wave and nearly spatially homogeneous distribution of MBSL.

**Figure 26:**
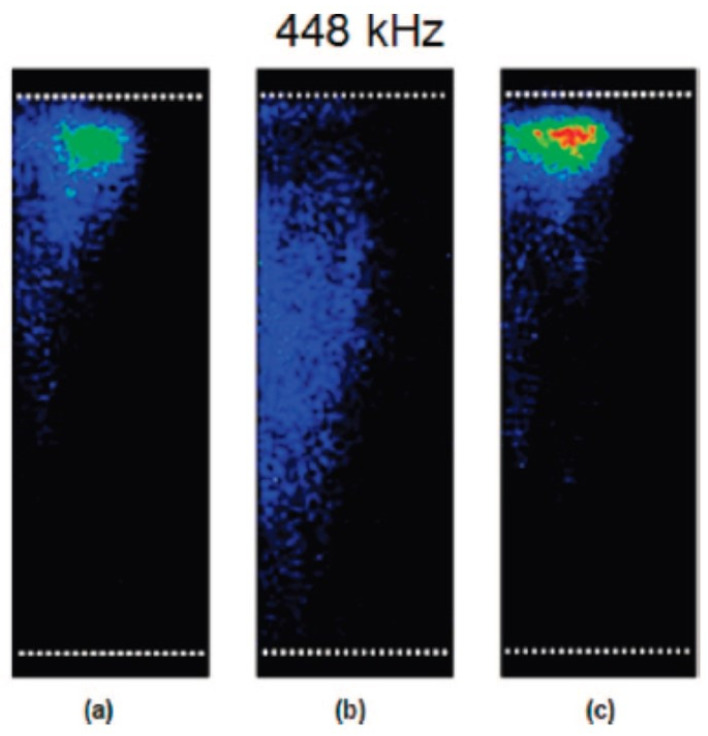
Images showing the effect of SDS on the MBSL structure generated at a frequency of 448 kHz and at a power of 1.1 W/cm^2^: (**a**) water, (**b**) 1 mM SDS, and (**c**) 10 mM SDS [59]. Exposure time was set to collect 2 × 10^5^ acoustic cycles. The white dotted lines above and below the MBSL structure denotes the liquid surface and the transducer position, respectively. The center axis of the vessel is located on the left side of the images. Copyright 2008, with the permission from American Chemical Society.

Addition of sodium dodecylsulfate (SDS), which is a surfactant, to the liquid water also changes the spatial distribution of MBSL intensity as shown in Figure 26 [59]. For 1 mM SDS solution, spatial distribution of MBSL intensity is nearly uniform as shown in Figure 26b. For 10 mM SDS solution, on the other hand, spatial distribution of MBSL intensity is localized near the liquid surface as shown in Figure 26c. For 1 mM SDS solution, there are very few visible large bubbles in the liquid because SDS strongly retards the bubble–bubble coalescence which is the main reason for the formation of visible large bubbles. For 10 mM SDS solution, on the other hand, electric negative charge of SDS on the bubble surface is neutralized by the excess number of positive ions, which results in the bubble–bubble coalescence under ultrasound and formation of visible large bubbles. As in the previous cases, Anderson localization of an acoustic wave due to visible large bubbles may be relevant to the spatial distribution of MBSL intensity in aqueous SDS solutions.

## 7. MBSL Quenching

**Figure 27:**
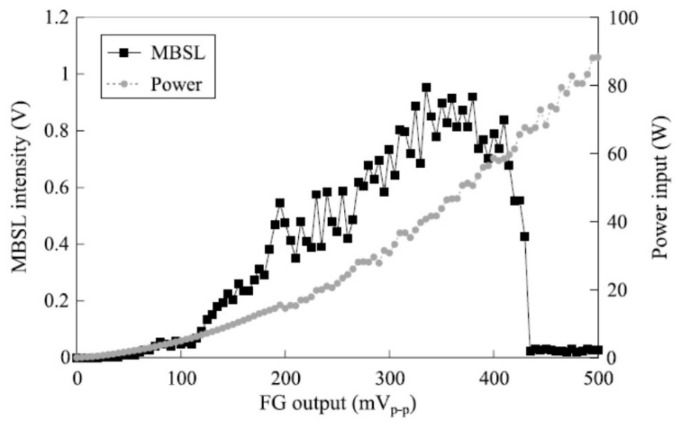
Dependence of MBSL intensity on FG output at 132.2 kHz and changes of power input to the transducer [68]. Reprinted from Ultrasonics, vol. 40, S. Hatanaka, K. Yasui, T. Kozuka, T. Tuziuti, and H. Mitome, Influence of bubble clustering on multibubble sonoluminescence, pp. 655–660, Copyright 2002, with the permission from Elsevier.

**Figure 28:**
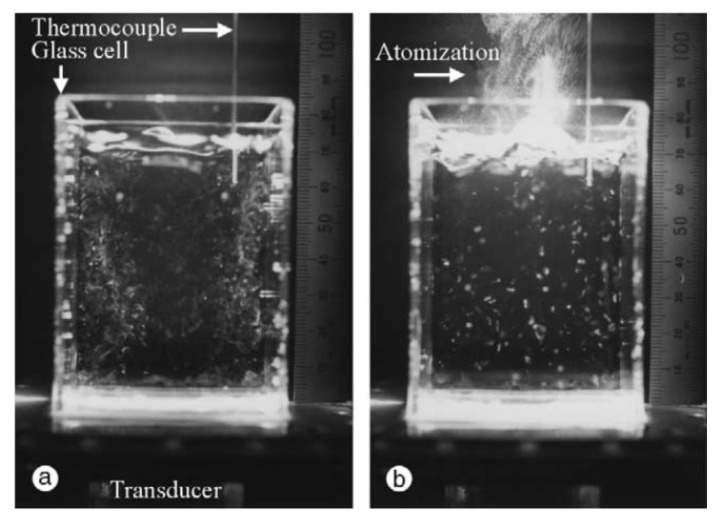
Photographs of cavitation bubbles at 132.2 kHz for the FG outputs of (**a**) 400 mV_p-p_ and (**b**) 450 mV_p-p_ taken with a still camera with 33 ms exposure [68]. Reprinted from Ultrasonics, vol. 40, S. Hatanaka, K. Yasui, T. Kozuka, T. Tuziuti, and H. Mitome, Influence of bubble clustering on multibubble sonoluminescence, pp. 655–660, Copyright 2002, with the permission from Elsevier.

**Figure 29:**
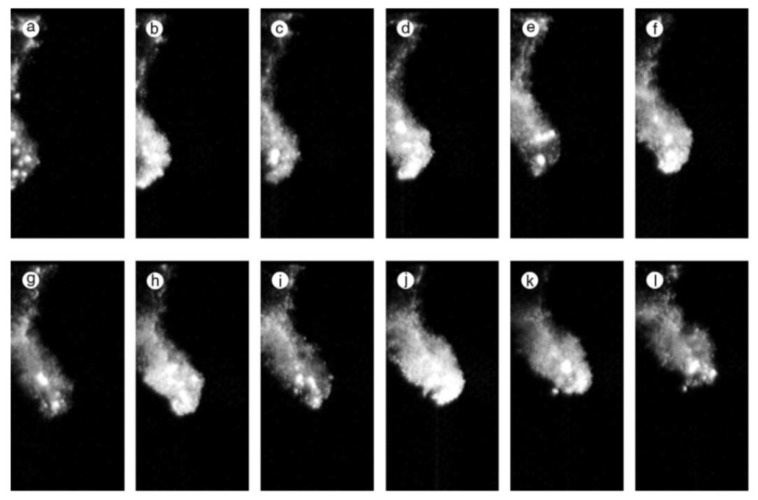
A bubble cluster observed at 23 kHz for the FG output of 1500 mV_p-p_ which is almost above the threshold for the MBSL quenching [68]. Copyright 2002, with the permission from Elsevier.

It has been experimentally reported that MBSL is almost completely quenched above the threshold acoustic power as shown in Figure 27 [68]. Initially, MBSL intensity increases as acoustic power increases. However, above the threshold, MBSL is almost completely quenched. There is a distinct difference between the cavitating liquid below and above the MBSL quenching threshold by visual observation as shown in Figure 28 [68]. Just below the MBSL quenching threshold shown in Figure 28a, most of the cavitation bubbles are seen near the side wall of the liquid container like smokes or filaments. On the other hand, above the MBSL quenching threshold shown in Figure 28b, white “particles” larger than 1 mm are seen almost spatially uniformly inside the liquid. The “particles” move around in the liquid. The “particles” are probably clusters of bubbles shown in Figure 29 [68]. Thus, the formation of the bubble clusters may be one of the reasons for the MBSL quenching. The bubble cluster is probably a dynamic object such that bubbles in the cluster frequently coalesce each other by secondary Bjerknes force and that the coalesced bubbles subsequently disintegrate into daughter bubbles [19]. It is suggested that bubbles in the bubble clusters are inactive in MBSL. Further studies are required on the dynamics of bubble clusters.

**Figure 30:**
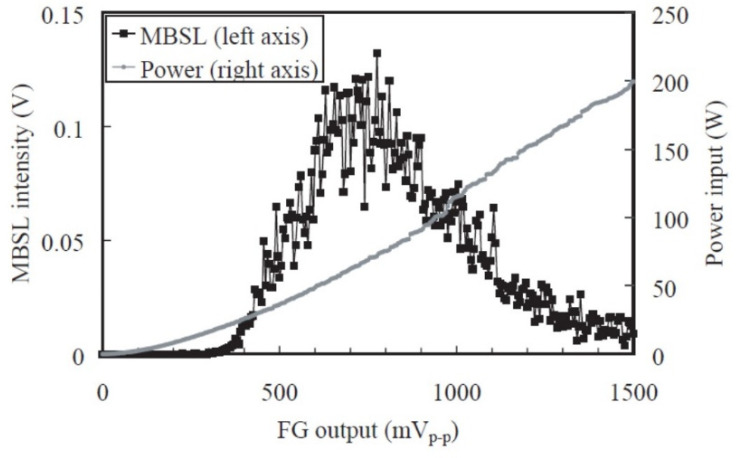
Dependence of MBSL intensity on FG output at 23 kHz and changes of power input to the transducers [69]. Copyright 2001, with the permission from IOP Publishing.

**Figure 31:**
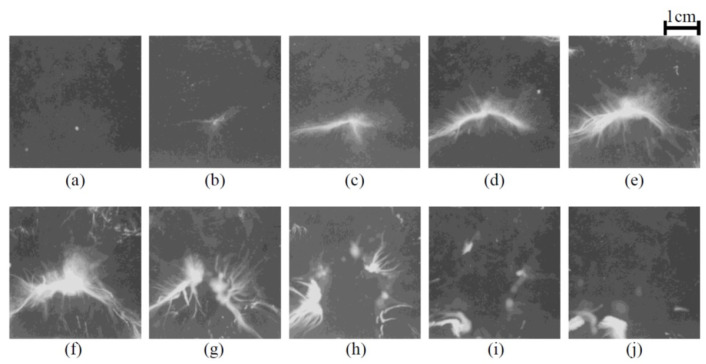
Photographs of cavitation bubbles taken with the still camera with the exposure of 2 ms at 23 kHz for the FG outputs of (**a**) 100, (**b**) 200, (**c**) 300, (**d**) 500, (**e**) 600, (**f**) 700, (**g**) 900, (**h**) 1000, (**i**) 1100, and (**j**) 1200 mV_p-p_, respectively [69]. Copyright 2001, with the permission from IOP Publishing.

**Figure 32:**
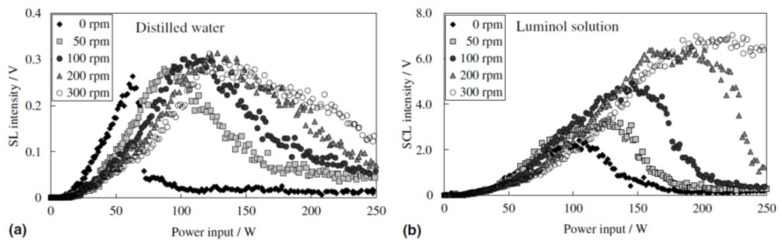
Dependence of intensities of sonoluminescence (SL) (**a**) and sonochemiluminescence (SCL) (**b**) on power input at 99 kHz for various rotational speeds of stirrer [70]. Copyright 2006, with permission from Elsevier.

Another mechanism for MBSL quenching is the repulsion of bubbles from pressure antinodes by primary Bjerknes force at excessive acoustic power as shown in Figure 30 and Figure 31 [5,44,45,69]. Initially, the MBSL intensity increases as acoustic power increases. It corresponds to the photographs of cavitation bubbles from (c) to (f) in Figure 31. From (g) to (j) in Figure 31, the MBSL intensity decreases as acoustic power increase as shown in Figure 30. As seen in Figure 31g–j, cavitation bubbles are gradually repelled from the pressure antinode as acoustic power increases. This is an important reason for the MBSL quenching. Indeed, by stirring the liquid, the MBSL quenching is considerably suppressed as shown in Figure 32a [70]. In Figure 32b, the quenching behavior is shown for SCL in aqueous luminol solution. Although the quenching of SCL and that of MBSL is similar, there is some difference between them probably because the threshold acoustic amplitude for SCL is smaller than that for MBSL [43,62]. Further studies are required on this topic.

## 8. Na-Line Emission

**Figure 33:**
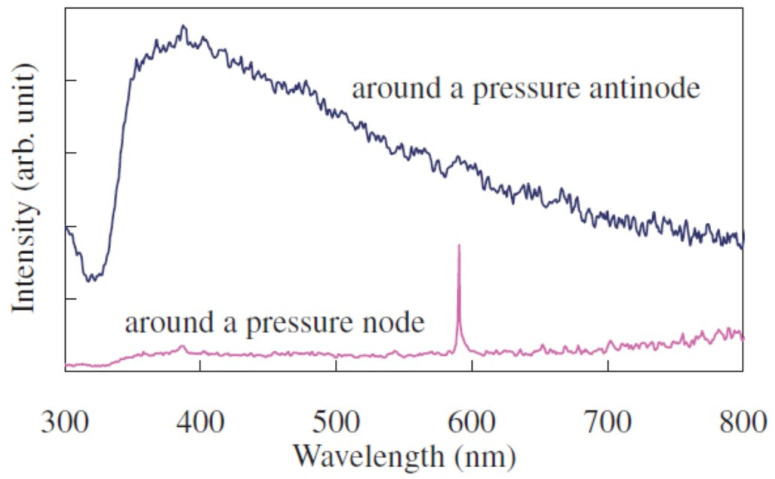
MBSL spectra in the orange region below the pressure node and the blue-white one around the pressure antinode [56]. Copyright 2010, The Japan Society of Applied Physics.

MBSL in aqueous solution containing Na^+^ ions often exhibits Na-line emission at 590 nm in wavelength as shown in Figure 1 as orange light [3,71]. As seen in Figure 1, the orange regions (Na-line emitting regions) are different from the blue regions (continuum emitting regions) as proved in Figure 33 [56]. In other words, cavitation bubbles which emit the Na-line are different from those which emit continuum component [72].

The Na-line emission in MBSL originate from the gas phase inside bubbles because the detailed structure of Na-line strongly depends on the gas species inside bubbles such as CO_2_ [3]. For the Na-line emission from the gas phase inside bubbles, Na atoms should enter bubbles by liquid jets because Na^+^ ions are nonvolatile. In other words, Na-line emitting bubbles are nonspherical jetting bubbles.

**Figure 34:**
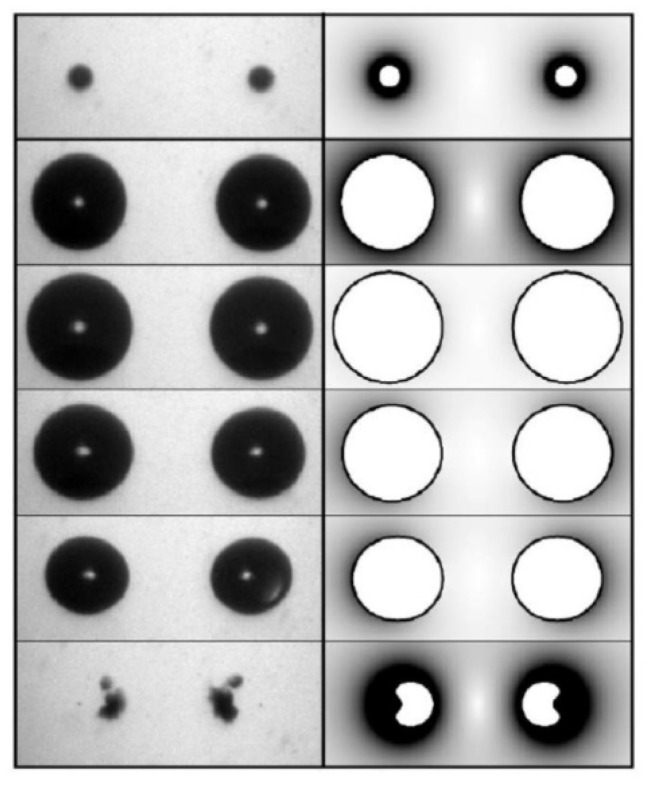
Comparison between experiment and simulation of the cavitation of two bubbles initially separated by 400 μm [73]. Copyright 2006, with the permission of AIP Publishing.

There are mainly three mechanisms in jetting of bubbles [5]. One is the simultaneous collapse of a pair of bubbles as shown in Figure 34 [73]. This is also called bubble–bubble interaction. The reason for the jetting is the difference of instantaneous local pressure between the outer and inner sides of the bubble surface. Another is the bubble collapse in a traveling-wave ultrasound [74]. In a traveling-wave ultrasound, the instantaneous acoustic pressure is different between ultrasound incoming and outgoing sides on the bubble surface. The other is the bubble collapse near a solid or liquid surface because the instantaneous local pressure is different between the solid or liquid surface side and the other side of the bubble surface [75,76,77].

Due to jetting, Na atoms enter bubbles. Furthermore, the bubble temperature decreases by jetting, which may result in weaker continuum emission from jetting bubbles [74]. Thus, Na-line-emitting bubbles (orange light) may not emit continuum component (blue light).

**Figure 35:**
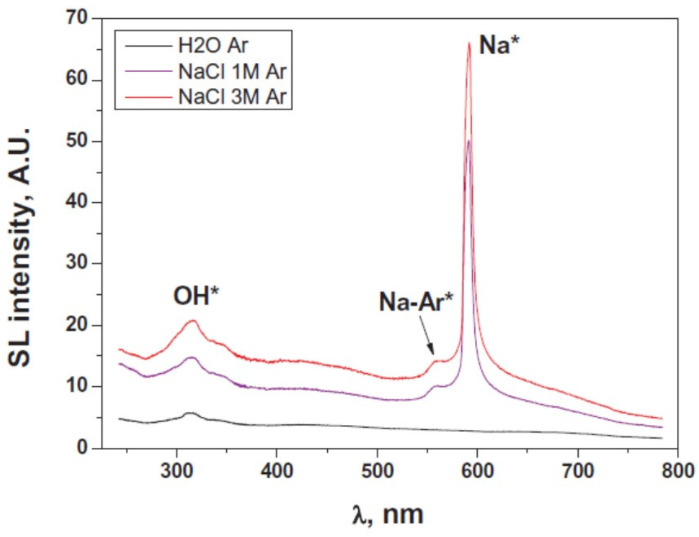
Measured MBSL spectra under Ar flow, in water and in 1 M and 3 M NaCl aqueous solutions (362 kHz, 43 W, 10 °C) [78]. Copyright 2019, with permission from Elsevier.

MBSL spectra from 1 M and 3 M NaCl aqueous solutions saturated with Ar are shown in Figure 35 as well as that from pure water saturated with Ar [78]. The continuum component of MBSL as well as OH-line emission in NaCl aqueous solutions is stronger than that from pure water. There are two factors on the role of the salt (NaCl). One is the retardation of bubble–bubble coalescence, which results in the increase in number of active bubbles and decrease in number of visible large bubbles [5,79]. The other is the decrease of gas solubility, which results in the decrease in the total amount of bubbles in the liquid and the increase in the standing-wave component of an ultrasonic wave [78]. Further studies are required on the mechanism of MBSL enhancement in NaCl aqueous solutions.

## 9. Ultrafine Bubbles

Cavitation threshold, which is the threshold acoustic amplitude for generation of cavitation bubbles, decreases by the presence of cavitation nuclei such as solid particles and tiny gas bubbles [5,80,81]. In other words, MBSL can be enhanced by the addition of an appropriate number of solid particles or tiny gas bubbles to the liquid [82].

Ultrafine bubbles or bulk nanobubbles are defined as gas bubbles smaller than 1 μm in diameter [83,84]. It is currently a hot topic because ultrafine bubbles are commercially used in bathing, cleaning, washing machines, plant cultivation, etc. [84] Ultrafine bubbles are usually produced by hydrodynamic cavitation such as using Venturi tube, swirling flow, injection of pressurized water containing gas, etc. [84] Initially, the liquid water is milky because it contains a lot of microbubbles. In a few minutes after stopping the production, most of microbubbles disappear at the liquid surface by buoyancy. Some of microbubbles are stabilized as ultrafine bubbles if the surface of a bubble is partly covered with hydrophobic impurities [85]. Ultrafine bubbles are stable for a few months or more [86]. They are confirmed as gas bubbles by their disappearance after freeze-thaw process [86].

Hata et al. [82] experimentally reported that MBSL intensity increased by the addition of ultrafine bubbles of about 100 nm in diameter with the number concentration of about 10^6^ mL^−1^. Further studies are required on this topic.

## 10. Brightest MBSL

MBSL from water saturated with air is very weak in intensity and very hard to see even in a dark room although it is easily observable by a photographic camera [62]. MBSL from water saturated with Ar is stronger in intensity and easier to see in a dark room [87]. However, it is still too weak to see in an illuminated room. On the other hand, MBSL from Xe in concentrated sulfuric acid (H_2_SO_4_) is so bright that it is observable with naked eyes even in an illuminated room [2,55,56,57].

Kappus et al. [88] experimentally reported that a Xe bubble in phosphoric acid (H_3_PO_4_), which has relatively low saturated vapor pressure, emitted 150 ns flash of broadband light that exceeded 100 W in peak intensity by the impact of a steel cylinder in which a Xe bubble was introduced against a solid steel base.

**Figure 36:**
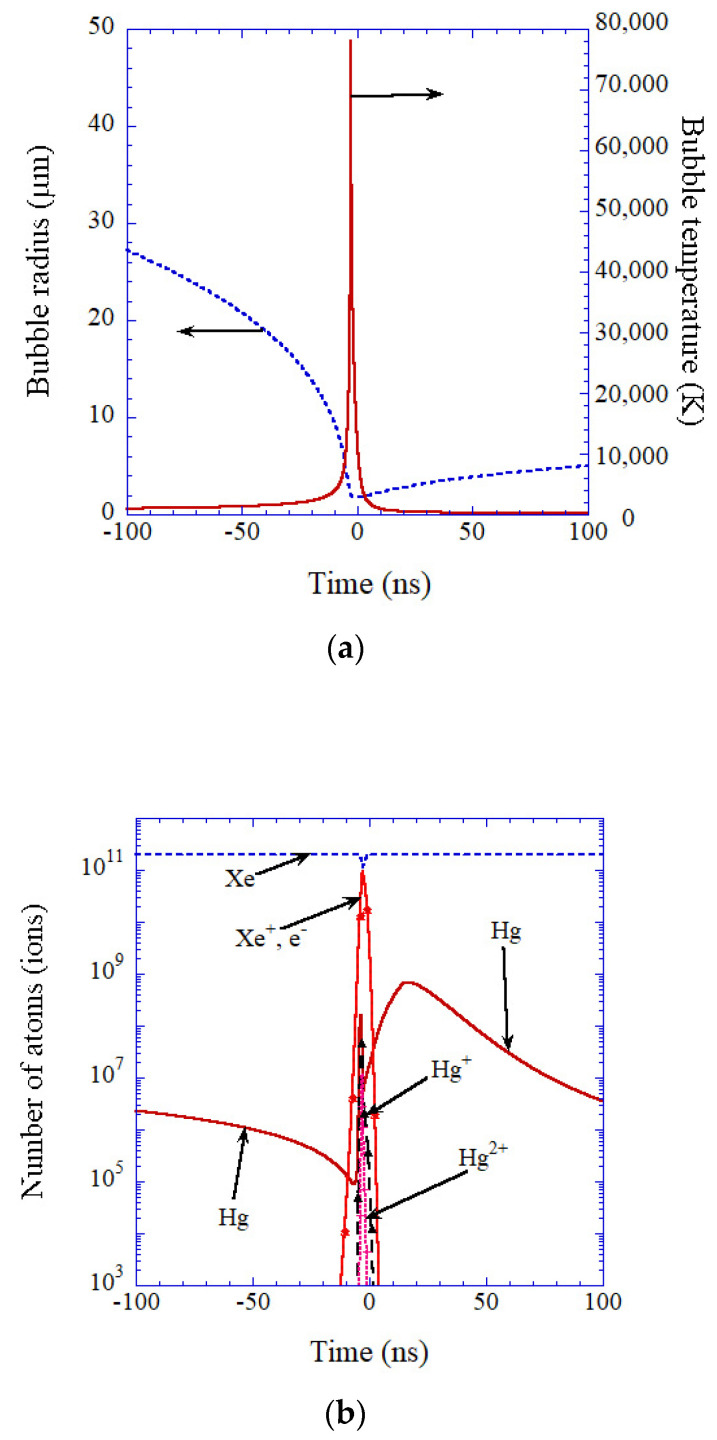

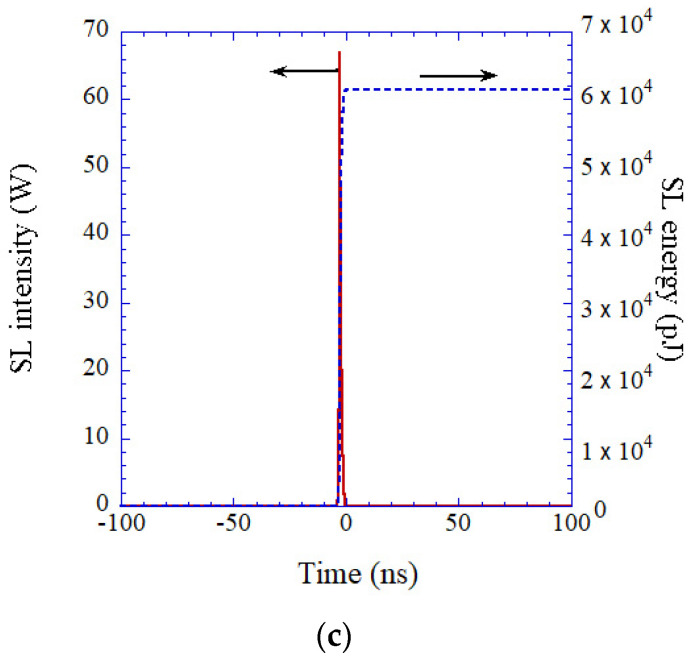
The result of numerical simulation for a Xe bubble in mercury as a function of time for 200 ns near the end of the bubble collapse (R_0_ = 10 μm, S = 10^5^ m^−1^) [42]. (**a**) The bubble radius (dotted line) and the temperature inside a bubble (solid line). (**b**) The number of atoms (ions) inside a bubble with logarithmic vertical axis. (**c**) The SL intensity (solid line) and its time integral (dotted line). Copyright 2012, with permission by the American Physical Society.

Numerically, the SL intensity is predicted to exceed 60 W from a Xe bubble in mercury, which has four orders of magnitude lower saturated vapor pressure than that of water, as shown in Figure 36 using the theoretical model described in Section 2 [42]. The bight SL from a Xe bubble is due both to lower ionization potential and lower thermal conductivity than those of other gases [42]. Lower ionization potential results in higher concentration of free electrons and consequently brighter SL due to stronger light emissions from plasma. Lower thermal conductivity results in higher bubble temperature and consequently the stronger light emissions from plasma.

MBSL intensity is determined not only by the light intensity from a bubble but also by number of active bubbles in SL. The number of active bubbles in MBSL has not yet been fully studied either experimentally or theoretically [89,90,91]. Further studies are required on the brightest MBSL.

## 11. Applications of MBSL

MBSL has been used to visualize an acoustic field through the spatial distribution of active bubbles in SL especially in studies on ultrasonic cleaning, sonochemical reactors, and medical applications such as cancer treatment using high-intensity focused ultrasound (HIFU) [59,60,61,62,92,93]. In the medical applications [92,93,94,95], MBSL is used to obtain the information on the existence of inertial cavitation bubbles (active bubbles in SL).

In sonodynamic therapy in which certain classes of drug can be activated by ultrasound, it has been suggested that MBSL light activates photosensitive drug [96,97]. Further studies are required on this topic.

From the spectra of MBSL, various information on the physical and chemical nature of the bubble interior can be obtained such as the bubble temperature and pressure, chemical species present inside a bubble, etc. [7,8,46,55,98,99,100,101,102,103]

Another interesting application of MBSL as well as SCL is installation art by modern artists [104].

## 12. New Development and Unsolved Problems

Cairos and Mettin [105] experimentally reported that bubbles that develop a liquid jet during collapse can flash intensely by high-speed recording in xenon saturated phosphoric acid. Yu et al. [106] performed numerical simulations of bubble collapse near a rigid wall and reported the spatial distribution of temperature inside a jetting bubble. Further studies are required on SL intensity from a jetting bubble including Na-line emission as well as its pulse width [107].

Lee and Choi [108] experimentally reported that a SBSL bubble is positively charged although a microbubble in the absence of ultrasound is negatively charged for pH > 4 [109,110,111]. They suggested that local pH near the wall of a SBSL bubble is lower than 4 because nitrite and nitrate ions are formed by chemical reactions of nitrogen inside a bubble. Further studies are required on this topic including polarization of the liquid under the pressure gradient (flexoelectric effect, which is different from that in solid crystals [112,113]) and temperature gradient (thermoelectric effect) [112].

Boyd et al. [114] numerically suggested that relatively strong UV light is radiated by the interaction of SL light with UV plasmon modes of the metal under the condition of aspherical air bubble collapse near a gallium-based liquid-metal microparticle. This may be applied to disinfecting water contaminated by pathogens such as bacteria and viruses. Further studies are required on this topic.

Fernandez Rivas et al. [115] reported MBSL from a microreactor. Further studies are required on this topic including peculiar bubble dynamics in microfluidics [116,117].

Numerical simulations discussed in Section 2, Section 3 and Section 4 are based on the theoretical model of a spherical isolated bubble. Further studies are required on numerical simulations by advanced models applicable to moving and extremely strongly interacting bubbles, nonspherical bubble collapse, splitting/merging processes, liquid sprays into the bubble, collective phenomena like change of speed of sound in a cavitating liquid, nonharmonic pressure fields, nonstationary parameters in the liquid by heating and degassing due to ultrasound irradiation, and uneven bubble distribution. Non-equilibrium plasma should also be considered [37]. In other words, these are the limitations of the theoretical model discussed in Section 2. Recently, Liang et al. [118] reported numerical simulations of SL spectra from two interacting bubbles.

## 13. Conclusions

Active bubbles in MBSL are classified into two categories. One is vaporous bubbles which are mostly filled with water vapor even at the end of the bubble collapse. The other is gaseous bubbles which are mostly filled with noncondensable gases such as air or argon at the end of the bubble collapse. MBSL from vaporous bubbles is by chemiluminescence of OH as well as emissions from weakly ionized plasma such as electron-atom bremsstrahlung. MBSL from gaseous bubbles is by emissions from plasma formed inside a bubble at the end of the collapse such as electron-atom bremsstrahlung and radiative recombination of electrons and positive ions. The plasma formation inside a bubble is due to high bubble temperature and the ionization potential lowering caused by the extremely high density inside a bubble at the end of the bubble collapse. Vaporous bubbles are seen at relatively high acoustic amplitudes at relatively low ultrasonic frequency. A SBSL bubble is a gaseous bubble because acoustic amplitude in SBSL is much lower than the threshold one for repulsion of the primary Bjerkens force.

MBSL has been used to visualize an acoustic field through spatial distribution of active bubbles in SL. Spatial distribution of MBSL is strongly influenced by the presence of visible large bubbles, which may be related to Anderson localization of an acoustic wave.

Orange light due to Na-line emission in aqueous solution containing Na^+^ ions originates in jetting bubbles by bubble–bubble interaction or by traveling-wave ultrasound.

The brightest MBSL may be from Xe bubbles in sulfuric or phosphoric acid which has relatively low saturated vapor pressure.

The application of MBSL in sonodynamic therapy is required to be studied further.
